# Japanese Clinical Practice Guidelines for Rehabilitation in Critically Ill Patients 2023 (J-ReCIP 2023)

**DOI:** 10.1186/s40560-023-00697-w

**Published:** 2023-11-07

**Authors:** Takeshi Unoki, Kei Hayashida, Yusuke Kawai, Shunsuke Taito, Morihide Ando, Yuki Iida, Fumihito Kasai, Tatsuya Kawasaki, Ryo Kozu, Yutaka Kondo, Masakazu Saitoh, Hideaki Sakuramoto, Nobuyuki Sasaki, Ryuichi Saura, Kensuke Nakamura, Akira Ouchi, Saiko Okamoto, Masatsugu Okamura, Tomoki Kuribara, Akira Kuriyama, Yujiro Matsuishi, Norimasa Yamamoto, Shodai Yoshihiro, Taisuke Yasaka, Ryo Abe, Takahito Iitsuka, Hiroyasu Inoue, Yuki Uchiyama, Satoshi Endo, Kazuki Okura, Kohei Ota, Takahisa Otsuka, Daisuke Okada, Kengo Obata, Yukiko Katayama, Naoki Kaneda, Mio Kitayama, Shunsuke Kina, Ryuichi Kusaba, Masanari Kuwabara, Naoki Sasanuma, Masahiro Takahashi, Chihiro Takayama, Naonori Tashiro, Junko Tatsuno, Takahiko Tamura, Mitsuhiro Tamoto, Asuka Tsuchiya, Yusuke Tsutsumi, Tadashi Nagato, Chihiro Narita, Tomohiro Nawa, Tadayoshi Nonoyama, Masatoshi Hanada, Kotaro Hirakawa, Akiko Makino, Hirotaka Masaki, Ryosuke Matsuki, Shinya Matsushima, Wataru Matsuda, Saori Miyagishima, Masaru Moromizato, Naoya Yanagi, Kota Yamauchi, Yuhei Yamashita, Natsuhiro Yamamoto, Keibun Liu, Yuki Wakabayashi, Shinichi Watanabe, Hiroshi Yonekura, Nobuto Nakanishi, Tetsuya Takahashi, Osamu Nishida, The Committee for the Clinical Practice Guidelines of Early Mobilization and Rehabilitation in Intensive Care of the Japanese Society of Intensive Care Medicine

**Affiliations:** 1 Department Acute and Critical Care Nursing, School of Nursing, Sapporo City University, Sapporo, Japan; 2 Department of Emergency Medicine, South Shore University Hospital, Northwell Health, Bay Shore, NY USA; 3 Department of Nursing, Fujita Health University Hospital, Toyoake, Japan; 4 Department of Clinical Practice and Support, Hiroshima University Hospital, Hiroshima, Japan; 5 Department of Pulmonary Medicine, Ogaki Municipal Hospital, Ogaki, Japan; 6 Faculty of Physical Therapy, School of Health Sciences, Toyohashi Sozo University, Toyohashi, Japan; 7 Department of Rehabilitation Medicine, Showa University School of Medicine, Tokyo, Japan; 8 Department of Pediatric Critical Care, Shizuoka Children’s Hospital, Shizuoka, Japan; 9 Department of Rehabilitation Medicine, Nagasaki University Hospital, Nagasaki, Japan; 10 Department of Emergency and Critical Care Medicine, Juntendo University Urayasu Hospital, Urayasu, Japan; 11 Department of Physical Therapy, Faculty of Health Science, Juntendo University, Tokyo, Japan; 12 Department of Critical Care and Disaster Nursing, Japanese Red Cross Kyushu International College of Nursing, Munakata, Japan; 13 Department of Rehabilitation Medicine, St. Marianna University School of Medicine, Kawasaki, Japan; 14 Department of Rehabilitation Medicine, Division of Comprehensive Medicine, Osaka Medical and Pharmaceutical University School of Medicine, Takatsuki, Japan; 15 Department of Critical Care Medicine, Yokohama City University Hospital, Yokohama, Japan; 16 Department of Adult Health Nursing, College of Nursing, Ibaraki Christian University, Hitachi, Japan; 17 Department of Nursing, Hitachi General Hospital, Hitachi, Japan; 18 Berlin Institute of Health Center for Regenerative Therapies (BCRT), Charité – Universitätsmedizin Berlin, Berlin, Germany; 19 Department of Primary Care and Emergency Medicine, Kyoto University Graduate School of Medicine, Kyoto, Japan; 20 School of Nursing, St. Luke’s International University, Tokyo, Japan; 21 Department of Nursing, Toyama Prefectural Central Hospital, Toyama, Japan; 22 Department of Pharmaceutical Services, Hiroshima University Hospital, Hiroshima, Japan; 23 Global Nursing Research Center, Graduate School of Medicine, the University of Tokyo, Tokyo, Japan; 24 Department of Rehabilitation, Saitama Medical Center, Jichi Medical University, Saitama, Japan; 25 Department of Rehabilitation, Amagasaki Daimotsu Rehabilitation Hospital, Amagasaki, Japan; 26 Department of Rehabilitation, Showa University School of Nursing and Rehabilitation Sciences, Yokohama, Japan; 27 Department of Rehabilitation Medicine, School of Medicine, Hyogo Medical University, Nishinomiya, Japan; 28 Rehabilitation Center, Amayama Hospital, Matsuyama, Japan; 29 Division of Rehabilitation, Akita University Hospital, Akita, Japan; 30 Department of Emergency and Critical Care Medicine, Graduate School of Biomedical and Health Sciences, Hiroshima University, Hiroshima, Japan; 31 Department of Rehabilitation Medicine, Okayama University Hospital, Okayama, Japan; 32 Department of Rehabilitation, Saiseikai Kumamoto Hospital, Kumamoto, Japan; 33 Department of Rehabilitation, Japanese Red Cross Okayama Hospital, Okayama, Japan; 34 Department of Nursing, Sakakibara Heart Institute, Fuchu, Japan; 35 Rehabilitation Division, Hokkaido Medical Center for Child Health and Rehabilitation, Sapporo, Japan; 36 Nursing Department, Kanazawa Medical University Hospital, Uchinada, Japan; 37 Department of Rehabilitation, Nakagami Hospital, Okinawa, Japan; 38 Department of Rehabilitation Medicine, Kyushu University Hospital, Fukuoka, Japan; 39 Department of Cardiology, Toranomon Hospital, Tokyo, Japan; 40 Department of Rehabilitation, Hyogo Medical University Hospital, Nishinomiya, Japan; 41 Department of Rehabilitation, Sapporo General Hospital, Sapporo, Japan; 42 Shironishi Hospital, Nagano, Japan; 43 Rehabilitation Center, Showa University Hospital, Tokyo, Japan; 44 Department of Nursing, Kokura Memorial Hospital, Kitakyusyu, Japan; 45 Department of Anesthesiology and Intensive Care Medicine, Kochi Medical School, Nankoku, Japan; 46 Department of Nursing, Kyoto University Hospital, Kyoto, Kyoto Japan; 47 Department of Emergency and Critical Care Medicine, Tokai University School of Medicine, Kanagawa, Japan; 48 Department of Emergency Medicine, National Hospital Organization Mito Medical Center, Mito, Japan; 49 Department of Respiratory Medicine and Infectious Diseases, JCHO Tokyo Yamate Medical Center, Tokyo, Japan; 50 Department of Emergency Medicine, Shizuoka General Hospital, Shizuoka, Japan; 51 Department of Pediatric Cardiology, Hokkaido Medical Center for Child Health and Rehabilitation, Sapporo, Japan; 52 Department of Rehabilitation, University of Fukui Hospital, Fukui, Japan; 53 Department of Rehabilitation, Sakakibara Heart Institute, Fuchu, Japan; 54 Department of Nursing, Nagoya University Hospital, Nagoya, Japan; 55 Department of Rehabilitation, Kansai Electric Power Hospital, Osaka, Japan; 56 Faculty of Health Science, Kyorin University, Mitaka, Japan; 57 Department of Emergency Medicine & Critical Care, Center Hospital of the National Center for Global Health and Medicine, Shinjuku, Japan; 58 Division of Rehabilitation, Sapporo Medical University Hospital, Hokkaido, Japan; 59 Department of Nursing, Chubu Tokushukai Hospital, Kitanakagusuku, Japan; 60 Department of Rehabilitation, Kitasato University Medical Center, Kitamoto, Japan; 61 Department of Rehabilitation, Steel Memorial Yawata Hospital, Kitakyushu, Japan; 62 Division of Rehabilitation Medicine, Gunma Prefectural Cardiovascular Center, Maebashi, Japan; 63 Department of Anesthesiology and Critical Care Medicine, Yokohama City University School of Medicine, Yokohama, Japan; 64 Critical Care Research Group, The Prince Charles Hospital, Chermside, QLD Australia; 65 Faculty of Medicine, The University of Queensland, Brisbane, QLD Australia; 66 Non-Profit Organization ICU Collaboration Network (ICON), Tokyo, Japan; 67 Department of Nursing, Kobe City Center General Hospital, Kobe, Japan; 68 Department of Physical Therapy, Faculty of Rehabilitation, Gifu University of Health Science, Gifu, Japan; 69 Department of Anesthesiology and Pain Medicine, Fujita Health University Bantane Hospital, Nagoya, Japan; 70 Department of Disaster and Emergency Medicine, Graduate School of Medicine, Kobe University, Kobe, Japan; 71 Department of Anesthesiology and Critical Care Medicine, School of Medicine, Fujita Health University, Toyoake, Japan; 72 Department of Physical Therapy Science, Nagasaki University Graduate School of Biomedical Sciences, Nagasaki, Japan

**Keywords:** Critically ill patient, Early rehabilitation, Intensive care, Guidelines

## Abstract

Providing standardized, high-quality rehabilitation for critically ill patients is a crucial issue. In 2017, the Japanese Society of Intensive Care Medicine (JSICM) promulgated the “Evidence-Based Expert Consensus for Early Rehabilitation in the Intensive Care Unit” to advocate for the early initiation of rehabilitations in Japanese intensive care settings. Building upon this seminal work, JSICM has recently conducted a rigorous systematic review utilizing the Grading of Recommendations, Assessment, Development, and Evaluation (GRADE) methodology. This endeavor resulted in the formulation of Clinical Practice Guidelines (CPGs), designed to elucidate best practices in early ICU rehabilitation. The primary objective of this guideline is to augment clinical understanding and thereby facilitate evidence-based decision-making, ultimately contributing to the enhancement of patient outcomes in critical care settings. No previous CPGs in the world has focused specifically on rehabilitation of critically ill patients, using the GRADE approach. Multidisciplinary collaboration is extremely important in rehabilitation. Thus, the CPGs were developed by 73 members of a Guideline Development Group consisting of a working group, a systematic review group, and an academic guideline promotion group, with the Committee for the Clinical Practice Guidelines of Early Mobilization and Rehabilitation in Intensive Care of the JSICM at its core. Many members contributed to the development of the guideline, including physicians and healthcare professionals with multiple and diverse specialties, as well as a person who had been patients in ICU. Based on discussions among the group members, eight important clinical areas of focus for this CPG were identified. Fourteen important clinical questions (CQs) were then developed for each area. The public was invited to comment twice, and the answers to the CQs were presented in the form of 10 GRADE recommendations and commentary on the four background questions. In addition, information for each CQ has been created as a visual clinical flow to ensure that the positioning of each CQ can be easily understood. We hope that the CPGs will be a useful tool in the rehabilitation of critically ill patients for multiple professions.

**Graphical Abstract:**

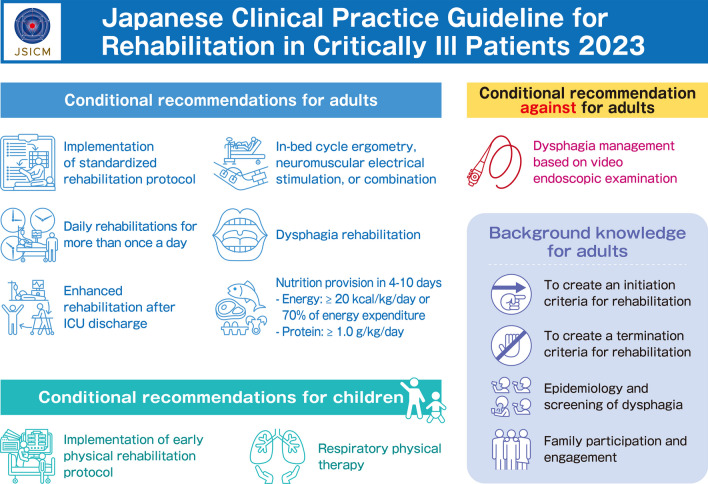

**Supplementary Information:**

The online version contains supplementary material available at 10.1186/s40560-023-00697-w.

## Introduction

Although advances in intensive care have increased the number of patients who can be saved, after being discharged from the hospital, those patients face a variety of problems that make it difficult for them to return to their daily lives. Rehabilitation for patients who had been critically ill is needed to prevent complications and facilitate the return to daily life of these individuals. In 2017, the Japanese Society of Intensive Care Medicine (JSICM) promulgated the “Evidence-Based Expert Consensus for Early Rehabilitation in the Intensive Care Unit” to advocate for the early initiation of rehabilitations in Japanese intensive care settings [1]. Building upon this seminal work, JSICM has recently conducted a rigorous systematic review (SR) utilizing the Grading of Recommendations, Assessment, Development, and Evaluation (GRADE) methodology. This endeavor resulted in the formulation of Clinical Practice Guidelines (CPGs), designed to elucidate best practices in early ICU rehabilitation. The primary objective of this guideline is to augment clinical understanding and thereby facilitate evidence-based decision-making, ultimately contributing to the enhancement of patient outcomes in critical care settings. In developing the CPGs, a Guideline Development Group (GDG), consisting of the Committee for the Clinical Practice Guidelines of Early Mobilization and Rehabilitation in Intensive Care of the JSICM, was established. Since multidisciplinary collaboration is extremely important in rehabilitation, the GDG for the CPGs was composed of members from multiple professions (physicians, physical therapists, nurses, occupational therapists, pharmacists, etc.). The physicians included not only intensivists but also a variety of specialists, such as rehabilitation physicians, emergency medicine physicians, and respiratory physician. In addition, working groups (WGs), which included physical therapists, nurses, physicians, occupational therapists, and a person who had been patients in ICU, were organized as an adjunct to the GDG. To promote the development of CPGs in an appropriate manner, an Academic Guideline Promotion Group was established to provide support from an academic perspective, from a neutral standpoint. To ensure quality and transparency of the work process, the committee conducted peer review within the committee, open discussions within each team, and solicited public comments.

Based on discussions among the group members, eight areas were identified as issues that require attention and were designated as areas of clinical importance. Fourteen clinical questions (CQs) were developed for each area of clinical importance. Then, 10 GRADE recommendations and four commentaries were provided as answers to the forward questions (FQs) and background questions (BQs), respectively. In addition, we created a clinical flow to allow the positioning of each CQ to be easily understood visually. We hope that the CPGs will contribute to effective clinical decision-making for multiple professions involving rehabilitation of critically ill patients. The J-ReCIP 2023 original Japanese version will be published in December 2023 in JSICM official journal the Journal of JSICM [2023; Volume 30(Supplement)]. The article was translated into English and published in the Journal of Intensive Care, the English-language journals of the society.

### Name

The official name of the guideline is “the Japanese Clinical Practice Guidelines for Rehabilitation in Critically Ill Patients 2023,” abbreviated to J-ReCIP 2023.

### Purpose

The purpose of the CPGs is to serve as a reliable guideline for rehabilitation of critically ill patients and to assist healthcare professionals in making appropriate decisions to improve the prognosis of these patients, as well as the quality of life (QOL) of the patients, their families, and care providers. At the same time, we also aim to identify future research questions.

### Target population

The target patient population includes patients entering the ICU, and their families and care providers. Patients in the process of recovery after discharge from the ICU, their families, and care providers are also included. Care providers in this guideline are assumed to be family members who provided care free-of-charge, rather than nurses, certified care workers, or other personnel. Patients and care providers included in this guideline are assumed to be adults, with the exception of those in some CQs.

### Perspectives covered by the clinical practice guidelines

In the CPGs, we consider recommendations from the standpoint of the individual patient, family member, and care provider, rather than the general public.

### Target users

The target healthcare professionals are not only those who work with patients and their families in the ICU, but also those who work in general wards and outpatient clinics after patients leave the ICU.

### Relationship with existing guidelines

Although several CPGs mention rehabilitation of critically ill patients, such as the Japanese Clinical Practice Guidelines for Management of Sepsis and Septic Shock [2] and the Japanese Guidelines for the Management of Analgesia, Sedation, and Delirium for Adult ICU Patients [3], no practice guidelines that specifically focus on the rehabilitation of critically ill patients are available. A related document, “Evidence based expert consensus for early rehabilitation in the intensive care unit” (2017) [1], by the JSICM, was available, although it is not CPGs. In the CPGs, we conducted SR, used the GRADE approach, and followed standard clinical practice guideline development methods to describe our recommendations.

### Organization

#### Guideline development group

The GDG consisted of 17 members: one director, who was in charge of the Committee for the Clinical Practice Guidelines of Early Mobilization and Rehabilitation in Intensive Care of the JSICM, one chairperson, two vice-chairs, 12 committee members, and one advisor. The policy was to include a wide variety of professionals involved in rehabilitation, which included intensivists, emergency physicians, rehabilitation physicians, nurses, physical therapists, and other professionals involved in the rehabilitation of critically ill patients. The GDG selected CQs and PICOs for the CPGs, voted on the recommended text, and responded to public comments.

The committee members served as group leaders and deputy group leaders of the WGs for each of the key clinical areas (hereinafter referred to as “areas”) described below, and with the assistance of the WGs described later, developed the CQs and PICOs, created the Evidence-to-Decision (EtD) table, drafted recommendations, and wrote the text. The advisors were positioned to provide overall advice and were not involved in specific tasks, including voting.

In addition, the group leader and deputy group leader of each area, who are members of the committee, did not conduct SR in their area of responsibility. However, they could communicate with the person who conducted the SR via the mailing list as appropriate. In such cases, the Academic Guideline Promotion Group (described below) monitored the content of the communication to ensure that the opinions of the committee members did not influence the methods or results of the SR.

#### Working groups

The role of WGs was to assist the group leaders of each area, who were the committee members. The WG members were selected through an open recruitment process and consisted of experts from various professions, who assisted the group leaders in preparing the CQs, PICOs, EtD tables, and recommendations, as well as involved in SR, if desired. Overall, 43 individuals were assigned, with an average of 2–3 individuals per CQ.

#### Systematic review group

Systematic review members were recruited from among the working groups and also publicly from the JSICM members.

#### Academic guideline promotion group

This group ensured in the entire process that the development of the CPGs was carried out in a smooth and appropriate manner. The group drafted, prepared, and distributed various materials, provided advice to the committee members, and conducted peer review. The group monitored the discussions on the mailing lists of all areas and provided advice as necessary to ensure that the discussions proceeded smoothly. The group also worked to ensure that SR was conducted appropriately by creating videos for those parts of the SR that were difficult to understand.

### Important clinical areas

For the important clinical areas, the projected CQs were brainstormed within the GDG and were categorized into eight areas. The following WGs were responsible for each area.

- WG1. Exercise therapy in ICU (including respiratory physiotherapy).
- WG2. Neuromuscular electrical stimulation and in-bed cycle ergometer.
- WG3. Dysphagia rehabilitation.
- WG4. Standards for mobilization.
- WG5. Nutritional therapy.
- WG6. Rehabilitation for critically ill children.
- WG7. Rehabilitation after ICU discharge.
- WG8. Family visitation and family participation in rehabilitation.

### Scope not covered by the clinical practice guidelines

Patients who did not require intensive care in the ICU were not covered.

### Ways to reflect the values of the target population (patients, families, and care providers)

To reflect the values of the patients and their families, one healthcare provider who had been admitted to an ICU due to a serious condition was added to one of the WGs. Care was taken to ensure that the other WGs could also ask the opinion of that member.

### Ensuring quality and transparency

The following activities were conducted to ensure quality and transparency.

1. Education was provided using videos and manuals to standardize knowledge among all the members.
2. Public comments were sought from the JSICM, as well as the Japanese Association of Rehabilitation Medicine, the Japanese Society of Physical Therapy, the Japan Academy of Critical Care Nursing, and the Japanese Society of Dysphagia Rehabilitation when drafting the CQs and recommendations.
3. Peer reviews of PICO, EtD tables, and texts were conducted anonymously by the committee members.
4. As a general rule, discussions within the WGs were conducted via the emailing list, and members of the Academic Guideline Promotion Group monitored discussions in all areas to identify and correct inappropriate methods and discussions. When teleconferences were held exceptionally, basically, the Academic Guideline Promotion Group was also involved, and the discussions were recorded, so that the discussions could be reviewed.
5. Declarations regarding conflicts of interest (COI) were made in accordance with the COI Management Guidelines of the Japanese Association of Medical Sciences, and COIs were confirmed by a group independent of the committee. Detailed information of COI and the roles in creating this guideline are summarized in Additional file 1.

### Drafting funds

This guideline was developed with funds from the JSICM. No compensation was received by any member in the preparation of these guidelines. No intention or interest of the medical society was reflected in the development of the recommendations.

### Strategies to disseminate the clinical practice guidelines

**Fig. 1:**
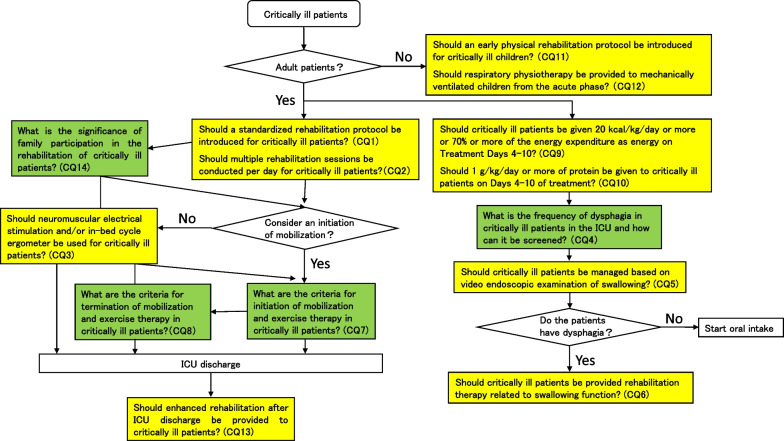
Clinical flow of the clinical practice guidelines

**Fig. 2:**
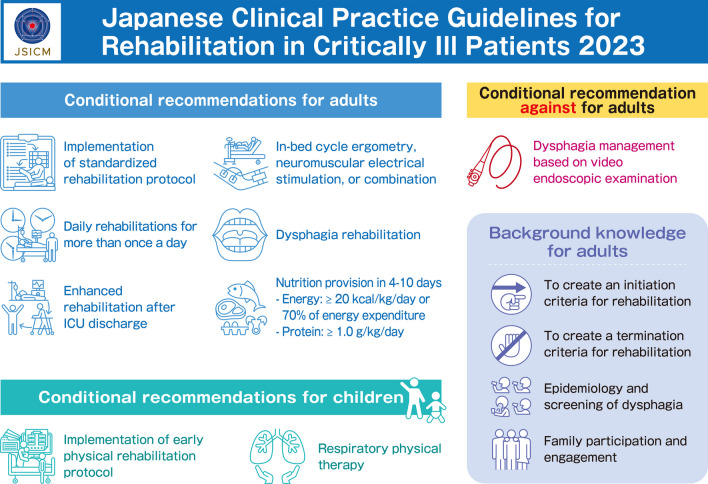
Graphical abstract of the clinical practice guidelines

A clinical flow (Fig. 1), graphic abstracts (Fig. 2), and digest versions of the CPGs were created for the convenience for users. We will also strive to raise awareness at scientific meetings, seminars, etc. Furthermore, the committee will monitor the status of guideline dissemination and will continue to improve the strategy for dissemination.

### Outline of clinical practice guidelines drafting method

This guideline was developed through three major processes: (1) CQ drafting; (2) SR search, collection of evidence, integration, and evaluation of certainty; and (3) formulation of recommendations. Relevant information for a recommendation based on GRADE were available at Additional file 2: Appendix 2.

#### CQ drafting

Based on the following rules for CQ drafting, CQ drafts were developed by the group leaders in the area with the assistance of the WG members and were approved by the committee after peer review by the committee members. After soliciting public comments from August 30 to September 10, 2021, the CQs were revised based on the received comments, after which 14 CQs were finalized by the committee.

#### Background questions

BQs are considered common knowledge, such as information written in textbooks. This guideline focuses on foreground questions (FQs) that contribute to decision-making. On the other hand, in some areas, the addition of BQs contributes to the dissemination of knowledge. The committee decided the policy on BQs that the number of BQs should not be minimized.

Draft recommendations for the finalized CQs were developed in each area, and after mutual peer review by the committee members, revisions were made until the committee reached an agreement rate of at least 90%, thus building consensus. Votes for and against the BQs were made by anonymous online voting. Voting for the BQ began on March 27, 2022, and was finalized on July 6, 2022.

#### Matters related to systematic review

##### Evidence search

Types of evidence: Only Randomized Controlled Trial (RCT) was included in the CPGs.

Database: Literature were obtained by electronic searches of MEDLINE (via PubMed), the Japan Medical Abstracts Society, and the Cochrane Central Register of Controlled Trials databases.

Basic search policy: The PICO format was used to search for literature The basic format was a combination of P and I, while C was also sometimes specified. O was not included in the search formula. Only papers published in English and Japanese were included, and those for which only abstracts were published were not included.

Target period of search: The search period was defined to cover papers registered in the database by October 18, 2021.

Method of evaluation and integration of evidence: The Cochrane RoB tool for RCTs (RoB 1.0) was used to assess bias risk in individual RCT studies.

#### Final coordination from recommendation drafting to publication and matters related to publication

##### Drafting of recommendations

The evidence profile based on the GRADE system was developed by the SR group. The committee members and WG members collaborated to develop the EtD table and draft recommendation text based on the evidence profile. Recommendations were determined by consensus of the committee as indicated below.

##### Voting on draft recommendation text

The RAND/UCLA method (modified Delphi method) [4] was used to reach consensus among the committee members. Each committee member independently evaluated the proposed recommendations and assigned a score from 1 to 9 (1: strongly disagree; 9: strongly agree) and provided comments. Voting was conducted online anonymously, and was tabulated by non-voting members of the Academic Guideline Promotion Group, who calculated the median, interpercentile range (IPR), interpercentile range adjusted for symmetry (IPRAS), and Disagreement Index (DI).

After the vote, a committee session (panel meeting) was held to reach a consensus.

##### Formulation of proposed recommendations

The strengths of recommendations indicated by the GRADE system are classified as recommended = “1”, suggested = “2”, not suggested = “3”, and not recommended = ”4″. The certainty of evidence was classified as follows: high = “A”, moderate = “B”, low = “C”, and very low = “D”.

##### The method for determining recommendations

For Median < 7.5 or DI ≥ 0.2, the committee discussed and revised the EtD and recommended text and re-voted. For Median ≥ 7.5 and DI < 0.2, if there were important comments from committee members, the committee discussed and, if necessary, revised the EtD and the recommended text, and then re-voted. If there were no important comments, the results of the vote were reviewed and consensus was reached. During the first vote on the draft recommendation, there were important comments on all CQs. Consensus was reached at subsequent committee meetings and a re-vote was held. After the re-vote, all recommendations were approved.

##### Main text drafting

The text was prepared by the WG members under the responsibility of the group leaders of each area. The draft text was revised by the committee until the approval rate reached 90% or more. All the main texts were approved on December 17, 2022.

##### Final adjustments

We invited public comments on the recommendations. An external assessment was conducted. The final version of the guidelines was finalized with reference to the above assessment.

### Plans for revision

These CPGs are to be revised every 4 years. The next revision is scheduled for 2027. The Intensive Care and Early Rehabilitation Committee will continue to monitor publications, and if important findings are published before the revision and the need for immediate revision arises, the Intensive Care and Early Rehabilitation Committee may discuss and revise the CPGs.

### External assessment

To evaluate the validity of this guideline, an external assessment was conducted by experts in methodology, the Japanese Association of Rehabilitation Medicine, and the Japanese Association for Acute Medicine experts. We revised this CPG based on experts’ evaluation.

### CQ1: Should a standardized rehabilitation protocol be introduced for critically ill patients?

Answer: We suggest the introduction of a rehabilitation protocol for critically ill patients (Grade 2D: Certainty of evidence = “Very low”).

#### Rationale

Early rehabilitation for critically ill patients has been reported to contribute to preventing muscle weakness and improving exercise capacity and activities of daily living (ADL). However, in clinical practice, the timing and content of rehabilitation varies among different facilities, leading to variability in its effectiveness. Nevertheless, initiating rehabilitation according to a standardized protocol can lead to benefits, such as reduced duration of mechanical ventilation, shorter ICU stay, and improved ADL. Therefore, investigating the effectiveness of implementing protocolized rehabilitation program in the ICU holds clinical significance. According to the results of our SR, 23 RCTs that met the PICO were included [5–27]. A meta-analysis was conducted using these studies. The estimated effect size for basic activities (6 RCTs; *N *= 595) showed a significantly higher standardized mean difference (SMD) of 0.62 (95% confidence interval [CI] 0.01 higher to 1.23 higher). For ADL (5 RCTs; *N *= 641), the estimated effect size was a significantly higher SMD of 0.15 (95% CI 0.27 lower to 0.57 higher). Regarding muscle strength (5 RCTs; *N *= 272), the estimated effect size was a significantly higher mean difference (MD) of 4.52 (95% CI 1.54 lower to 10.59 higher). As for the duration of mechanical ventilation (16 RCTs; *N *= 1165), the estimated effect size was a significantly shorter MD of 1.28 days (95% CI 1.68 days shorter to 0.87 days shorter). Finally, for ICU length of stay (19 RCTs; *N *= 1838), the estimated effect size indicated a significantly shorter MD of 1.53 days (95% CI 2.3 days shorter to 0.77 days shorter). Rates of delirium during ICU stay were not reported. The SMDs for fundamental activities and ADLs were small. However, all reported outcomes favored the intervention group, and beneficial effects were judged as “moderate”. Conversely, the estimated effect size for all adverse events (7 RCTs; *N *= 994) was 24 fewer events per 1000 individuals (95% CI 61 fewer to 71 more), indicating a “trivial” undesirable effect. Based on these findings, the positive effects were deemed “moderate,” the negative effects were considered “trivial,” and the recommendation learned toward favoring the intervention.

### CQ2: Should multiple rehabilitation sessions be conducted per day for critically ill patients?

Answer: We suggest conducting multiple daily rehabilitation sessions for critically ill patients (GRADE 2D: Certainty of Evidence = “Very Low”).

#### Rationale

Early rehabilitation for critically ill patients has been reported to contribute to the prevention of muscle weakness and improving exercise capacity and ADL. The significance of early rehabilitation in this population has been increasingly recognized in recent years. Furthermore, although it is based on small-scale studies, some studies research has indicated that conducting multiple daily rehabilitation sessions (two or more times) enhances the Medical Research Council sum score (MRC-SS) after ICU admission [28]. However, there is no definitive consensus on the effectiveness of multiple daily rehabilitation sessions. Therefore, studying the effectiveness of conducting multiple daily rehabilitation sessions in the ICU holds clinically significant. A systematic review identified eight RCTs that matched the PICO criteria [22, 26, 28–33] and a meta-analysis was conducted using these studies. The estimated effect size for basic activities (1 RCT, *N *= 216) was an MD of 3.00 higher (0.33 higher to 5.67 higher), while for ADLs (2 RCTs, *N *= 204), the estimated effect size was an SMD of 0.22 higher (0.05 lower to 0.5 higher). As for muscle strength (2 RCTs, *N *= 87), the estimated effect size was an MD of 2.17 lower (5.62 lower to 1.29 higher). Furthermore, the estimated effect size for duration of mechanical ventilation (6 RCTs, *N *= 291) was an MD of 2.26 days shorter (3.86 days shorter to 0.65 days shorter), and for ICU length of stay (7 RCTs, *N *= 533) the estimated effect size was an MD of 2.24 shorter days (4.02 shorter to 0.46 shorter). There were no reported rates of delirium during ICU stay. Clinically significant outcomes, such as fundamental and ADL, duration of mechanical ventilation, and ICU length of stay, favored the intervention group. Conversely, muscle strength demonstrated superiority in the control group, but its significance was deemed lower compared to fundamental activities and ADL, and its overall impact on the effects was considered minimal. Although the SMD/MD for basic activities and ADL were small, the beneficial effects were categorized as “moderate.” Conversely, the estimated effect size for all adverse events (3 RCTs, *N *= 422) was more than 10 individuals per 1000 individuals (95% CI 10 fewer to 20 more), indicating a “trivial” adverse effect. Based on these findings, the beneficial effects were categorized as “moderate,” the adverse effects as “trivial,” and the recommendation was “probably in favor of the intervention.”

### CQ3: Should neuromuscular electrical stimulation and/or in-bed cycle ergometer be used for critically ill patients?

Answer: We suggest performing neuromuscular electrical stimulation for critically ill patients (GRADE 2B: Certainty of evidence = “Moderate”).

We suggest performing in-bed cycle ergometer (GRADE 2D: Certainty of evidence = “Very low”).

We suggest performing both neuromuscular electrical stimulation and in-bed cycle ergometer (GRADE 2B: Certainty of evidence = “Moderate”).

#### Rationale

ICU patients commonly experience muscle weakness due to accelerated protein catabolism associated with severe conditions, such as sepsis [34], which can occur early during their ICU admission. This muscle weakness not only leads to a decline in ADLs and exercise tolerance after ICU discharge, but also has the potential to impact mortality rates [35]. Early rehabilitation of these patients has been recommended to prevent and improve these conditions, although there is no consensus on the specific intervention methods. Neuromuscular electrical stimulation and in-bed cycle ergometer exercises are interventions that can be implemented early, regardless of the patient's level of consciousness or depth of sedation are expected to have beneficial effects on preventing muscle weakness and improving ADLs and exercise tolerance after ICU discharge. The clinical significance of evaluating the effectiveness of adding neuromuscular electrical stimulation and/or in-bed cycle ergometer exercises, in addition to standard early rehabilitation or no intervention, is substantial, and it was deemed an important clinical issue to be addressed in this guideline.

In the SR, we identified 19 RCTs on neuromuscular electrical stimulation [36–54], 10 RCTs on in-bed cycle ergometer [31, 54–62], and four RCTs on use of both neuromuscular stimulation and in-bed cycle ergometer [29, 54, 63, 64] that met the PICO criteria. We, therefore, conducted a meta-analysis using these reports.

Regarding neuromuscular electrical stimulation, the estimated effect sizes for ADL (Barthel Index) (2 RCTs; *N *= 106) was an MD of 10.76 points higher (95% CI 12.95 points lower to 34.48 points higher). For the MRC-SS (2 RCTs; *N *= 68), the estimated effect size was an MD of 4.68 points higher (95% CI 2.66 points lower to 12.03 points higher). The estimated effect size for muscle mass (2 RCTs; *N *= 42) was an MD of 0.37 mm higher (95% CI 2.57 mm lower to 3.30 mm higher). For the duration of mechanical ventilation (10 RCTs; *N *= 502), the estimated effect size was an MD of 1.0 day shorter (95% CI 2.18 days shorter to 0.18 days longer). The estimated effect size for length of hospital stays (7 RCTs; *N *= 411) was an MD of 3.77 days shorter (95% CI 7.98 days shorter to 0.43 days longer). There were no reported RCTs regarding the 6-min walk distance (6MWD). All outcomes favored the intervention, and the desirable effect was deemed “moderate.” The estimated effect size for all adverse events (4 RCTs; *N *= 139) was 140 fewer events per 1000 individuals (95% CI 380 fewer to 100 more), indicating a “trivial” undesirable effect. Given the moderate desirable effect and trivial undesirable effect, we determined “favors the intervention”.

Regarding in-bed cycle ergometer, the estimated effect size for the 6MWD (1 RCTs; *N *= 67) was an MD of 53.0 m longer (95% CI 16.85 m shorter to a 122.85 m longer). For the MRC-SS, the estimated effect size (2 RCTs; *N *= 110) was an MD of 0.19 points lower (95% CI 2.91 points lower to 2.53 points higher). The estimated effect size for muscle mass (1 RCTs; *N *= 24) was an MD of 2.75 mm higher (95% CI 4.17 mm lower to 9.67 mm higher). The estimated effect size for duration of mechanical ventilation (7 RCTs; *N *= 319) was an MD of 0.76 days longer (95% CI 0.69 days shorter to 2.2 days longer). The estimated effect size for length of hospital stays (6 RCTs; *N *= 277) was an MD of 1.28 days shorter (95% CI 5.44 days shorter to 2.88 days longer). There were no reported RCTs regarding ADL. Each outcome shows a different direction, but considering the highest importance placed on the 6MWD, which favored the intervention, the desirable effect was deemed “small.” The estimated effect size for all adverse events (1 RCT; *N *= 67) was 0 fewer events per 1000 individuals (95% CI 60 more to 60 fewer), indicating a “trivial” undesirable effect. Given that the desirable effect was “small”, and the undesirable effect was “trivial,” we determined "probably favors the intervention”.

For use of both neuromuscular electrical stimulation and in-bed cycle ergometer, the estimated effect sizes for ADL (Katz Index and Barthel Index) (2 RCTs; *N *= 250) was an SMD of 0.21 higher (95% CI 0.29 lower to 0.71 higher). The estimated effect size for the 6MWD (1 RCT; *N *= 46) was an MD of 81.0 m longer (95% CI 7.01 m longer to 154.99 m longer). For the MRC-SS (3 RCTs; *N *= 477), the estimated effect size was an MD of 0.47 points higher (95% CI 4.09 points lower to 5.04 points higher). The effect size for muscle mass (3 RCT; *N *= 585) was an SMD of 0.39 higher (95% CI 0.13 higher to 0.65 higher). The estimated effect size for duration of mechanical ventilation (2 RCT; *N *= 474) was an MD of 0 days shorter (95% CI 0.25 days shorter to 0.25 days longer). For length of hospital stays (2 RCT; *N *= 301), the estimated effect size was an MD of 1.96 days shorter (95% CI 3.32 days shorter to 0.6 days shorter). The intervention group was found to be superior in most outcomes, and the desirable effect was assessed as “moderate”. The estimated effect size for all adverse events (1 RCT; *N *= 312) was 10 fewer events per 1000 individuals (95% CI 60 fewer to 30 more), indicating a “trivial” undesirable effect. Given the moderate desirable effect and trivial undesirable effect, we determined “favors the intervention”.

### CQ 4: What is the frequency of dysphagia in critically ill patients in the ICU and how can it be screened?

Answer: The exact frequency of dysphagia in critically ill patients in the ICU remains uncertain. Due to variations in practices and food cultures among countries, various screening methods have been devised for dysphagia, and no international standardization has been established. In addition, even if patients can swallow voluntarily, they may still experience silent aspiration, which makes it necessary to combine multiple screening methods to determine the presence of dysphagia (Provision of information for background question).

#### Background and importance of this CQ

In critically ill patients, swallowing function is often impaired due to interventions, such as the placement of a tracheal tube, tracheostomy, and surgical procedures. In particular, in older adults, pre-existing dysphagia may be present due to comorbidities and aging. Therefore, it is important to understand the frequency of dysphagia in critically ill patients. Dysphagia can also impact restrictions in oral intake, changes in dietary methods, decisions regarding discharge to home, and prognosis. Therefore, swallowing function should be evaluated at an appropriate time during ICU admission. Swallowing function assessment consists of screening to identify dysphagia and diagnostic swallowing function tests. However, the optimal screening methods and timing of tests for swallowing function in critically ill patients remain unknown. Various methods for evaluating swallowing function exist, but ICU patients are often attached to multiple medical devices and have limited mobility, which restricts the swallowing function tests that can be performed in the ICU setting. Given the above, dysphagia should not be overlooked in critically ill patients and the appropriate screening methods and timing of tests for this purpose should be clarified. Therefore, these issues were addressed as a CQ in this guideline.

#### Rationale

The frequency of dysphagia among critically ill patients in the ICU varies widely, due to the lack of standardized evaluation methods and diagnostic criteria, differences in study populations in previous studies, and variations in the timing of assessments. In recent years, various systematic reviews and cohort studies on dysphagia in the intensive care setting have been reported. An endoscopic evaluation of swallowing function in patients aged 65 years and older, who underwent more than 48 h of mechanical ventilation and subsequent extubation revealed dysphagia in 52% of the participants (22 of 42 individuals) [65]. In a systematic review examining the impact of laryngeal injury due to endotracheal intubation, dysphagia immediately after extubation was observed in 49% of cases (157 of 319 individuals) [66]. Among these cases, screening based on a swallowing score in 59 mechanically ventilated patients (with a mean intubation duration of 9.4 ± 6.1 days) revealed dysphagia in 57% of the 44 patients when they were assessed within 24 h after extubation. Furthermore, among the 15 patients assessed more than 24 h after extubation, 60% showed signs of dysphagia [66]. In another systematic review, dysphagia at 48 h after extubation was reported in at least 20% of patients with acute respiratory failure, and in cases requiring prolonged mechanical ventilation exceeding 48 h, this prevalence ranged from 50% to 60% [67]. Furthermore, in cardiac surgery patients who underwent more than 48 h of mechanical ventilation, 51% (130 of 254 individuals) had dysphagia during post-extubation swallowing assessments, with a higher frequency observed in cases with prolonged mechanical ventilation [68]. In a large cohort study involving 2,484 critically ill patients, 84% (374 out of 446 individuals) of ICU admissions who underwent screening were reported to have dysphagia [69]. However, since screening was not conducted in more than 60% of the study population, the actual frequency remains unknown. Based on the aforementioned information, it can be inferred that early dysphagia following mechanical ventilation in critically ill patients in the ICU occurs in approximately 50% of cases, although the specific screening methods and timing may vary. Furthermore, the prevalence of dysphagia is thought to increase with prolonged duration of mechanical ventilation.

Dysphagia in critically ill patients admitted to the ICU is associated with outcomes, such as the development of pneumonia, reintubation, length of hospital stays, home discharge rates, swallowing function at discharge, ability to resume oral intake, and in-hospital mortality rates [69]. Therefore, swallowing function assessments should be performed as early as possible after ICU admission and rehabilitation therapy should be initiated. Screening methods can be used early on to identify patients suspected of having dysphagia, which enables further examination, diagnosis, and treatment [70]. In Japan, hospital meals often include rice porridge, and emphasis is placed on food viscosity. However, in evaluations conducted in other countries, firm bread is included as an essential evaluation item in some cases [71]. Due to variations in circumstances and dietary cultures among countries, various methods have been devised, making it difficult to achieve international standardization. ICU patients are often attached to multiple medical devices, and their mobility is restricted. Thus, screening methods that can be easily performed at the bedside without requiring special equipment are ideal. Furthermore, these methods should demonstrate high validity, reliability, sensitivity, and specificity, and therefore, they need to be compared with gold standard techniques, such as video endoscopic examination of swallowing (VE) or video fluoroscopic swallowing studies (VF). The following are screening methods that are considered appropriate for dysphagia in critically ill patients admitted to the ICU. Since silent aspiration can occur even if patients can swallow voluntarily, other tests and symptoms may be combined to make an assessment.

#### Assessment of the morphology and function of the oral cavity and pharynx

Prior to conducting various screening methods, an assessment of the oral and pharyngeal morphology and function, as well as a neurological examination, should be performed. First, during observation of autonomous opening and closing movements, the oral cavity should be checked for contamination and tongue coating, and oral care should be provided. Next, the remaining teeth, loose teeth, and dental caries should be identified to assess chewing function. Swallowing function can be inferred by examining tongue movement, tongue deviation and atrophy during tongue protrusion, soft palate elevation, and the curtain sign. Facial nerve function, including the depth of the nasolabial groove, pursing of the lips, and corner of the mouth retraction, can affect the intraoral retention of the food bolus. In the evaluation of voice quality and articulation, breathy voice and reduced volume may suggest impaired glottal closure and possible nerve paralysis should be considered. In addition, wet hoarseness can be caused by the presence of airway secretions adhering to the vocal folds, but caution should be exercised, as this can also occur due to the pooling of saliva in the vallecula epiglottic and pyriform sinuses or due to penetration into the larynx.

### CQ5: Should critically ill patients be managed based on video endoscopic examination of swallowing?

Answer: We suggest against managing critically ill ICU patients based on VE (GRADE 2D: Certainty of evidence = “Very low”).

#### Rationale

Critically ill patients in the ICU often experience dysphagia due to factors, such as prolonged inability to take food orally, resulting in oral functional decline, the presence of an oral endotracheal tube, and systemic catabolism due to invasive procedures [69]. Swallowing function evaluation using VE is convenient as it allows direct visualization of the pharynx and larynx, making it highly applicable in ICU settings. However, the effectiveness of management based on VE in the ICU setting remains unclear.

In the SR, only one RCT that met the PICO criteria was identified [72]. No reports regarding outcomes, such as mortality, eating status, duration from extubation to oral intake, QOL, duration of hospital stay, or adverse events (excluding pneumonia and choking) were identified. Therefore, the desirable effects of the intervention were deemed “uncertain” due to a lack of evidence.

In the identified RCT (*N *= 70), the estimated effect size for reintubation was 24 more cases per 1000 people (ranging from 25 fewer to 539 more). The occurrence of pneumonia was set as a desirable effect, but based on one RCT (*N *= 70), the estimated effect size for pneumonia was 75 more cases per 1000 people (ranging from 33 fewer to 590 more), an undesirable effect suggesting an increase with the intervention. Therefore, the undesirable effect was considered to be “small” in magnitude.

In this CQ, the important outcomes examined were the occurrence of pneumonia and adverse events (reintubation) only. Since the intervention tended to increase harm, the control treatment was considered to be superior.

### CQ6: Should critically ill patients be provided rehabilitation therapy related to swallowing function?

Answer: We suggested that rehabilitation therapy related to swallowing function should be provided for critically ill patients (GRADE 2C: Certainty of evidence = “Low”).

#### Rationale

In critically ill patients in the ICU, swallowing function can be compromised due to factors, such as oral functional decline, placement of an oral endotracheal tube, tracheostomy, and a history of highly invasive procedures. Compromised swallowing function increases the risk of aspiration pneumonia and necessitates changes in the method of food intake in daily life, resulting in a deterioration of the patient's QOL. Rehabilitation therapy targeting oral and swallowing function is provided with the aim of improving swallowing function in these critically ill patients. However, the effectiveness of such rehabilitation interventions remains unclear.

In the SR, we identified 11 RCTs that met PICO criteria, which were included in a meta-analysis [73–83]. The estimated effect size for mortality (9 RCTs; *N *= 591) was 1 event fewer per 1000 individuals (ranging from 55 fewer to 95 more), but differences were not statistically significant. The estimated effect size for the incidence of pneumonia (5 RCTs; *n*= 500) was 145 events fewer per 1000 individuals (ranging from 196 to 80 fewer), suggesting a clinically significant effect. No reports addressed QOL or ADL. The estimated effect size for eating status (Functional Oral Intake Scale) (3 RCTs; *N *= 141) showed an MD of 0.79 higher (ranging from 0.21 lower to 1.79 higher), indicating a beneficial direction of the effect of the intervention. While a reduction in pneumonia incidence was observed, no clear effects were demonstrated for other outcomes, leading to the judgment of a “small” desirable effect.

The outcomes related to adverse events (4 RCTs; *N *= 419) showed an estimated effect size of 13 fewer events per 1000 individuals (ranging from 263 fewer to 574 more). Although the outcome was set as a harmful effect, a beneficial direction of the effect was observed, although without statistical significance, leading to a judgment of a “trivial” undesirable effect.

In this CQ, while a decrease in pneumonia incidence was observed, no other clear effects were identified. However, considering identification of a trivial undesirable effect, it was judged that the intervention was likely to be superior.

### CQ7: What are the criteria for initiation of mobilization and exercise therapy in critically ill patients?

#### Answer

After confirming the recovery or stabilization of their condition from a life-threatening crisis, initiation of mobilization and exercise therapy is considered in critically ill patients. No unified consensus on the criteria and timing for safe and effective initiation of mobilization and exercise therapy has been established. A comprehensive judgment should be made by the healthcare team by referring to the “Proposed Criteria for Initiation of Mobilization and Exercise Therapy in Critically Ill Patients” (Table 1) (Provision of information for background question).

**Table 1:** Proposed criteria for initiation of mobilization and exercise therapy in critically ill patients

Category	Item/Index	Acceptance criterion value or condition
Subjective symptoms	Pain	(If self-reported) NRS ≤ 3 or VAS ≤ 30 mm
		(If not self-reported) BPS ≤ 5 or CPOT ≤ 2
		No intolerable pain/agony
	Fatigue	No intolerable fatigue
	Dyspnea	No sudden dyspnea
Neurological	Sedation, agitation (RASS)	-2 ≤ RASS ≤ + 1 (If there is not enough staff for safety management) RASS + 2 is acceptable
	Consciousness level (GCS and JCS)	Opens eyes when called upon
Respiratory	Respiratory rate	5 breaths per minute ≤ RR ≤ 40 breaths per minute
	Percutaneous arterial oxygen saturation (SpO_2_)	SpO_2_ ≥ 88% or ≥ 90%
	Fraction of inspiratory oxygen (F_I_O_2_)	F_I_O_2_ < 0.6
	Positive end-expiratory pressure (PEEP)	PEEP < 10 cmH_2_O
	Guidelines for mechanical ventilation management	Not set for lung rest
Cardiovascular system	Heart rate	40 bpm ≤ HR ≤ 130 bpm
	Systolic blood pressure	90 mmHg ≤ sBP ≤ 180 mmHg
	Mean arterial pressure	60 mmHg ≤ MAP ≤ 100 mmHg
	Dose of hypertensive drug	No recent new drug initiation or dose increase prior to the start
	Arrhythmia	No arrhythmia that could disrupt the hemodynamics
	Myocardial ischemia	No ECG changes suggestive of new myocardial ischemia or no untreated myocardial ischemia
Device	Device and catheter	Insertion site is properly immobilized
Other	Intracranial pressure	ICP < 20 mmHg and no recent increase in ICP prior to start
	Body temperature	BT < 38.5ºC Not undergoing hypothermia therapy
	Bleeding	No active bleeding Hemoglobin concentration ≥ 7 g/dL
	Skeletal system	No unstable fractures
	Cerebrovascular event	No cerebrovascular events within 24 h
	Thromboembolism	Thromboembolism is under control
	Organ ischemia	No new-onset or uncontrolled organ ischemia

Initiation of mobilization or exercise therapy requires the consent of patient or their families, and the use of the initiation criteria is based on obtaining consent from the patient or their families

BT, body temperature; BPS, behavioral pain scale; CPOT, critical-care pain observation tool; F_I_O_2_, inspired oxygen fraction; ICP, intracranial pressure; NRS, numeric rating scale; RASS, Richmond agitation–sedation scale; RR, respiratory rate; VAS, visual analogue scale; PEEP, positive end-expiratory pressure; HR, heart rate; sBP, systolic blood pressure; MAP, mean arterial pressure; BT, body temperature; GCS, Glasgow coma scale; JCS, Japan coma scale

*In the context of this CQ, the term “mobilization and exercise therapy in critically ill patients” refers specifically to activities, such as getting out of bed and engaging in exercise therapy. It does not encompass activities, such as positioning, range of joint motion exercises performed in bed, neuromuscular electrical stimulation, or swallowing rehabilitation. Furthermore, when initiating rehabilitation based on the proposed criteria for initiation of mobilization and exercise therapy in critically ill patients, consent must be obtained from the patient or their family.

### CQ8: What are the criteria for termination of mobilization and exercise therapy in critically ill patients?

Answer: It is important to establish criteria for discontinuing mobilization and exercise therapy* in critically ill patients, as it is possible to destabilize their condition during the process. However, no consensus on the criteria for discontinuing mobilization and exercise therapy in critically ill patients has been established to date. The “Proposed Criteria for Discontinuation Mobilization and Exercise Therapy in Critically Ill Patients” (Table 2) may be used in accordance with facility capabilities or can be tailored to the specific disease or physiological state (Provision of information for background question).

**Table 2:** Proposed criteria for discontinuation of mobilization and exercise therapy in critically ill patients

Category	Item/index	Acceptance criterion value or condition	Remarks
Subjective symptoms	Pain, agony	Intolerable pain/agony	
	Fatigue	Intolerable fatigue	
	Dyspnea	Sudden dyspnea	
Neurological system	Consciousness level (GCS or JCS)	Reduced consciousness level compared to the start	
	Facial expression	Distress, pallor of the face, and appearance of cyanosis	
	Sedation (RASS), agitation	RASS ≤ − 3 or 2 < RASS Dangerous behaviors due to restlessness	
	Voluntary movement of limbs	Limb weakness	
Respiratory system	Respiratory rate	RR < 5 breaths per minute or RR > 40 breaths per minute	Excludes temporary condition
	Arterial blood oxygen saturation	SpO2 < 88% or < 90%	SpO2 < 88% in cases with poor oxygenation Excludes temporary condition
	Breathing patterns	Sudden increase in inspiratory or expiratory efforts (increased activity of neck accessory muscles, such as sternocleidomastoid, concavity of supraclavicular fossa, contraction of abdominal muscles, etc.)	Evaluation should include assessment of airway obstruction using techniques, such as auscultation
	Mechanical ventilation	Non-synchronicity that does not improve even after changes in ventilation settings	
		Bucking	Evaluate improvements by removal of airway secretions through aspiration
		Risk of accidental extubation or unplanned extubation of the endotracheal tube	
Cardiovascular system	Heart rate	HR < 40 bpm or HR > 130 bpm	If there is a significant decrease or increase in heart rate, even if it does not meet the criteria for discontinuation, discontinuation of mobilization or exercise therapy should be considered, and a physician should be consulted Excludes temporary condition
	Systolic blood pressure	sBP < 90 mmHg or sBP > 180 mmHg	If mobilization or exercise therapy has been initiated in a manner deviating from the criteria values on the left, the discontinuation criteria should be established as a team, including the physician. Similarly, if there is significant drop or rise in blood pressure without meeting the discontinuation criteria, mobilization or exercise therapy should be suspended, and a physician should be consulted Excludes temporary condition
	Mean arterial pressure	MAP < 60 mmHg or MAP > 100 mmHg	
	ECG findings	New arrhythmia requiring treatment Suspected myocardial ischemia	
Device	Device and catheter	Risk of catheter removal (or removal itself) Catheter insertion site bleeding Decreased flow rate	
Other	Patient refusal or discontinuation appeal		
	Characteristics of drainage	Suggested active bleeding	
	Surgical wound condition	Wound dehiscence	
	Sweating (hyperhidrosis), cold sweats		

ECMO, extracorporeal membrane oxygenation; IABP, intra-aortic balloon pumping; RASS, Richmond agitation–sedation scale; RR, respiratory rate; RRT, renal replacement therapy; VAD, ventricular-assist device; HR, heart rate; sBP, systolic blood pressure; SpO_2_, arterial blood oxygen saturation; MAP, mean arterial pressure; GCS, Glasgow coma scale; JCS, Japan coma scale

*In the context of the present CQ, the term “mobilization and exercise therapy in critically ill patients” refers specifically to activities, such as getting out of bed and engaging in exercise therapy. It does not encompass activities, such as positioning, range of joint motion exercises performed in bed, neuromuscular electrical stimulation, or swallowing rehabilitation. Furthermore, when discontinuing rehabilitation based on the proposed criteria for discontinuation of mobilization and exercise therapy in critically ill patients, consent must be obtained from the patient or their family.

### CQ9: Should critically ill patients be given 20 kcal/kg/day or more or 70% or more of the energy expenditure as energy on Treatment Days 4–10?

Answer: We suggest administering 20 kcal/kg/day or 70% or more of the energy expenditure as energy on treatment days 4–10 in critically ill patients (GRADE 2D: Certainty of evidence = “Very low”).

### Rationale

Previously, the target energy intake for critically ill patients has been recommended as 25–30 kcal/kg/day [84]. However, the formula used for this estimation was developed based on energy expenditure outside the acute phase [85, 86], and whether this is truly optimal for critically ill conditions remains unclear. Moreover, opinions vary depending on the timing, particularly in the early acute phase (the first 2–3 days after ICU admission) when it overlaps with the peak of invasiveness and inflammation, necessitating cautious nutrition delivery [87]. Against this backdrop, it is often challenging to achieve the target intake throughout the entire acute phase in actual clinical practice [88]. Given the essential need for a combination of appropriate nutritional therapy with early mobilization, this CQ evaluated the impact of energy intake on the outcomes of critically ill patients during days 4–10 of treatment, excluding the early acute phase. The evaluation focused on administering an energy intake of 20 kcal/kg/day or more, or providing at least 70% of the calculated energy expenditure. Considering the need for an adequate nutritional therapy combination, particularly in terms of energy provision for rehabilitation, this is crucial, as it can affect outcomes, such as ADL, physical function, and muscle mass. Therefore, this study addressed the importance of this issue in the context of rehabilitation for critically ill patients.

Based on our SR, 15 RCTs that matched the PICO criteria were identified from 18 published papers [89–106], and a meta-analysis was conducted using these studies. No RCTs reporting on ADL or changes in muscle mass were identified. For physical function (2 RCTs; *N *= 192), the estimated effect size, measured as the MD, was 0.58 higher (ranging from 4.77 lower to 5.92 higher). For health-related QOL (HRQOL) scores (2 RCTs; *N *= 551), the estimated effect size was 0.01 higher (ranging from 0.03 lower to 0.05 higher). While a trend for intervention showing superiority was found in terms of physical function and QOL outcomes, the effect sizes were small, indicating only trivial desirable effects. Regarding adverse events, the estimated effect size for diarrhea (3 RCTs; *N *= 1114) was 36 more cases per 1000 individuals (ranging from 9 fewer to 93 more). Considering the occurrence of adverse events, the undesirable effects were also considered to be trivial. Therefore, neither the intervention nor the control was deemed superior.

### CQ10: Should 1 g/kg/day or more of protein be given to critically ill patients on days 4–10 of treatment?

Answer: We suggest administering protein of 1 g/kg/day or more to critically ill patients on days 4–10 of treatment (GRADE 2D: Certainty of evidence “Very low”).

### Rationale

Similar to energy requirement, the optimal amount of protein to provide critically ill patients has not yet been clearly established. Protein is necessary for immune function [107] and tissue formation [108], but protein administration during the acute phase may also be associated with harm [109]. Moreover, opinions vary depending on the stage of administration, particularly regarding the early phase of acute illness (the first 2–3 days), where cautious protein administration is believed to be necessary due to the overlap with invasive procedures and the peak of inflammation [87]. To maintain and increase physical function and muscle mass, which are the goals of rehabilitation, appropriate protein administration is essential [110]. Therefore, in this guideline, the impact of administering protein at a dosage of 1 g/kg/day or higher on outcomes for critically ill patients during the treatment period from days 4 to 10, excluding the early acute phase, was evaluated. As such, we recognized it as an important clinical question in the context of rehabilitation for critically ill patients, as it can impact rehabilitation-related outcomes.

Based on the SR, 15 RCTs from 16 papers that met the PICO criteria were identified [100, 111–125], and were used for a meta-analysis. The desirable effects observed were as follows: for ADL outcomes (3 RCTs; *N *= 236), the estimated effect size was an MD of 21.55 higher (ranging from 1.3 lower to 44.4 higher). For physical function outcomes (2 RCTs; *n*= 65), the estimated effect size was an MD of 1 lower (ranging from 5.79 lower to 3.79 higher). For muscle mass changes (3 RCTs, *N *= 286), the estimated effect size was an SMD of 0.47 higher (ranging from 0.24 higher to 0.71 higher). Finally, for HRQOL scores (3 RCTs, *N *= 713), the estimated effect size was an SMD of 0.13 points lower (ranging from 0.31 points lower to 0.06 points higher). The estimated effect size for the adverse event of diarrhea (7 RCTs; *N *= 465) was 45 fewer events per 1000 individuals (ranging from 176 fewer to 140 more cases). The outcomes of ADL, muscle mass changes, and adverse events favored the intervention group, while the outcomes of physical function and HRQOL favored the control group. However, considering the low importance level assigned to these outcomes, the desirable effects were judged to be “small.” No significant increase in the occurrence of undesirable effects was observed, and therefore, the undesirable effects were judged to be “trivial.” With desirable effects being “small” and undesirable effects being “trivial,” the intervention was deemed to be likely to be superior.

### CQ11: Should an early physical rehabilitation protocol be introduced for critically ill children?

Answer: We suggest an early physical rehabilitation protocol be introduced for critically ill children (GRADE 2D: Certainty of evidence = “Very low”).

### Rationale

In the field of pediatric intensive care, knowledge is lacking regarding the effectiveness and safety of implementation of a physical rehabilitation protocol soon after ICU admission, with the goals of early mobilization and muscle strength preservation. However, similar to the adult settings, some pediatric patients struggle with mobilization and muscle recovery, even during the convalescence phase. Such patients may benefit from early physical rehabilitation after admission, leading to reduced risk of mortality and shorter hospital stays. On the other hand, concerns such as unplanned removal of various tubes and a lack of understanding among the healthcare staff may create hesitation regarding implementation of physical rehabilitation during the acute phase in critically ill children. Therefore, we considered that it would be important to investigate the effectiveness and safety of early physical rehabilitation in this CQ.

The results of the SR identified two relevant RCTs meeting the PICO criteria [126, 127], and a meta-analysis was performed on these studies. The estimated effect size for length of hospital stay (1 RCT; *N *= 58) was a mean difference (MD) of 0 days (95% CI 4.98 days shorter to 4.98 days longer), while the estimated effect size for length of ICU stay (1 RCT; *N *= 58) was an MD of 1.5 days longer (95% CI 2.63 days shorter to 5.63 days longer). No RCTs reporting on mortality were identified. Based on the above, even though the length of ICU stay was prolonged; because of the limited sample size and the inability to evaluate the outcome of mortality, the desirable effects of early physical rehabilitation were considered “unknown.” On the other hand, the estimated effect size for adverse events requiring therapeutic intervention (2 RCTs; *N *= 88) was 66 fewer events per 1000 individuals (95% CI 91 fewer to 151 more). Consequently, the intervention does not appear to increase adverse events, and therefore, the undesirable effects of early physical rehabilitation were considered “trivial.” In this CQ, while the undesirable effects were “trivial,” the desirable effects could not be determined. Therefore, the judgment of superiority between the intervention and control is “unknown” in this CQ. Nevertheless, considering that early physical rehabilitation protocols have already been implemented without additional resources in many hospitals and that adverse events are unlikely to increase, we decided to emphasize potential desirable effects of early physical rehabilitation protocols for critically ill children.

It should be kept in mind, however, that the effectiveness of implementing an early physical rehabilitation protocol for critically ill children could not be clearly determined in this study. Therefore, the effects should be evaluated individually for each patient.

### CQ12: Should respiratory physiotherapy be provided to mechanically ventilated children from the acute phase?

Answer: We suggest performing respiratory physiotherapy to mechanically ventilated children from the acute phase. (GRADE 2D: Certainty of evidence = “Very low”).

### Rationale

Children, particularly infants, are prone to developing atelectasis, because their high lung chest wall compliance is not elastic enough to counteract the recoil of the lungs, and also because their airways are narrow. Therefore, it has been reported that respiratory physiotherapy is actively performed in the acute phase of mechanical ventilation management [128]. However, comprehensive evidence is limited on whether respiratory physiotherapy accelerates ventilator liberation or ensures safety during the procedures. Therefore, we considered it important to examine this CQ.

The results of the SR identified two RCTs meeting the PICO criteria [129, 130], and a meta-analysis was performed using these studies. Both RCTs evaluated the effects of prone positioning intervention as compared to supine positioning as a control during the mechanical ventilation. The estimated effect size for mortality (2 RCTs; *N *= 143) was a risk difference (RD) of 107 fewer events per 1000 people (95% CI 178 fewer to 64 more), while the estimated effect size for duration of mechanical ventilation (1 RCT; *N *= 42) was a mean difference (MD) of 10.1 h longer (95% CI 37.57 h shorter to 57.77 h longer). No RCTs regarding lengths of hospital stay or ICU stay were identified. Based on these results, the desirable effect of respiratory physiotherapy was judged to be “moderate.” On the other hand, the estimated effect size for adverse events requiring therapeutic intervention (1 RCT; *N *= 101) was an RD of 22 fewer events per 1000 patients (95% CI 78 fewer to 175 more). Based on these results, it was considered that the intervention did not lead to an increase in adverse events, and therefore, the undesirable effect of respiratory physiotherapy was judged to be “trivial.” Considering the moderate desirable effect and the trivial undesirable effect in this CQ, the balance of effects is likely to favor the intervention.

It would be important to evaluate the effects of the intervention on individual patients carefully, keeping in mind that the two selected RCTs evaluated the effects of prone positioning management specifically, and no RCTs evaluating other interventions in respiratory physiotherapy were obtained.

### CQ13: Should enhanced rehabilitation after ICU discharge be provided to critically ill patients?

Answer: We suggest providing enhanced rehabilitation to critically ill patients after ICU discharge. (GRADE 2D: Certainty of evidence = “Very low”).

### Rationale

The objective of rehabilitation for critically ill patients is to maintain and improve ADL and to enhance QOL. Similar to rehabilitation in ICU, enhanced rehabilitation after ICU discharge is also performed with the goal of improving ADL, enhancing QOL, and facilitating social reintegration. However, no definitive consensus on the effects and adverse events associated with enhanced rehabilitation after ICU discharge has been established to date. It has been observed that physical, cognitive, and psychological impairments in critically ill patients persist for a long time, even after ICU discharge. Therefore, clarifying the effectiveness of enhanced rehabilitation after ICU discharge for critically ill patients is important, to develop appropriate rehabilitation plans for them. Our SR identified 20 RCTs that met the PICO criteria [131–150], which were included in a meta-analysis. The estimated effect size for QOL (physical) (9 RCTs; *N *= 807) was an SMD of 0.10 higher (95% CI 0.06 lower to 0.25 higher). For QOL (mental) (9 RCTs; *N *= 803), the estimated effect size was an SMD of 0.19 higher (95% CI 0.03 lower to 0.42 higher), and for QOL (overall) (5 RCTs; *N *= 424), the estimated effect size was an SMD of 0.22 higher (95% CI 0.09 lower to 0.54 higher). For ADL (2 RCTs; *N *= 115), the estimated effect size was an SMD of 0.41 lower (95% CI 1.28 lower to 0.46 higher). No RCTs reported on the effect of enhanced rehabilitation on return to work. Among the outcomes, the three clinically important QOL measures favored enhanced rehabilitation, while ADL favored comparison. With lower importance assigned to ADL than to QOL, the desirable effect was considered “small.” For all adverse events (4 RCTs, *N *= 166), the estimated effect size was 49 more events per 1000 individuals (95% CI 8 fewer to 310 more), with 0 events reported in both the intervention and comparison groups in three of four RCTs. Regarding the initially anticipated beneficial outcome on mortality (2 RCTs; *n*= 288), the estimated effect size was 22 more events per 1000 individuals (95% CI 31 fewer to 218 more), indicating a “small” undesirable effect. Considering that the importance assigned to mortality and all adverse events was lower than that assigned to QOL, the judgment was that “probably favors the intervention”.

### CQ14: What is the significance of family participation in the rehabilitation of critically ill patients?

Answer: The involvement of family in the rehabilitation of critically ill patients includes direct participation and assistance in actual rehabilitation activities, such as mobilization, providing encouragement by holding hands, assisting with ADLs and contributing to the patient’s comfort. By involving family members, the patient’s motivation for rehabilitation can be maintained, and is likely to have positive effects, such as reducing anxiety, discomfort, and post-rehabilitation fatigue. In addition, it can satisfy the needs of family members who wish to help the patient and can potentially improve negative beliefs, feelings of futility, and powerlessness. However, family involvement in rehabilitation may also impose psychological burdens on both the patient and the family. It is necessary to provide sufficient explanation and education to the family before suggesting their participation (Provision of information for background question).

#### Background and importance of this CQ

Critically ill patients are in a severe physical and mental condition, and the presence of family is crucial. Strategies commonly used in the routine care of critically ill patients, such as the ABCDEF bundle, incorporate elements of early rehabilitation and family involvement, which are considered important throughout the recovery process [151]. Previous studies have reported the needs of family members, including their desire to be involved in the patient’s care and to be given specific roles [152–155]. At present, clear definitions or specific methods for family involvement in the rehabilitation of critically ill patients are lacking, making it difficult to assess its effectiveness. However, the participation of family members in rehabilitation, which includes assisting with ADL and providing care that contributes to the patient's comfort, holds the potential to benefit both the patient and the family. For this reason, it has been addressed as a CQ in this guideline.

#### Rationale

Family-centered care is an approach to healthcare that respects the individual needs and values of each family and involves providing information, involving them in care, and supporting their decision-making processes [156]. In the context of rehabilitation for critically ill patients, the aspect of family involvement in rehabilitation as part of family-centered care has been considered, in addition to the mental and physical support for the patient [153]. Family participation in the rehabilitation of critically ill patients has been reported to include various activities, such as massage, passive and active exercises for limbs, positioning and turning, respiratory rehabilitation, early mobilization, including transfers and walking, and ADL practices, such as grooming. Family members have been reported to participate in these rehabilitation activities either alone or in collaboration with healthcare professionals [152–154, 157]. In addition, acts such as holding the patient’s hand for encouragement and wiping away sweat during rehabilitation to maintain the patient’s motivation have also been identified as possible ways for family members to participate in the rehabilitation process [152].

Although intervention studies examining the effects of family participation in rehabilitation for critically ill patients are limited, it has been shown to help maintain the patient’s motivation for rehabilitation and to reduce anxiety, discomfort, and post-rehabilitation fatigue [152, 153]. In addition, family involvement in providing care that contributes to the patient's comfort, such as massage, and assisting with positioning adjustments, has been associated with improvement of post-traumatic stress disorder symptoms in family members by 90 days after the patient’s discharge or death [157]. Approximately 70% of patients perceive family participation in their rehabilitation favorably, and over 80% of families express a desire to participate [155]. Family members often show interest in the patient’s medical condition and care, and they want to contribute to ensuring the best possible care for the patient [152]. Reports have indicated that involving family members in rehabilitation or providing them with psychological support roles can meet these family needs [152].

On the other hand, family participation may potentially cause psychological distress for both patients and family members. Some patients may wish to maintain their self-image or feel embarrassed about their appearance, while others may have concerns about the safety of family participation, questioning whether family members are skilled in providing care; hence, it is necessary to consider the patients’ emotions and social backgrounds when making these choices. Family members also express concerns about the potential negative impact on the patient's health or about interfering with healthcare professionals’ work [154]. In particular, positioning adjustments and mobilization assistance require specialized knowledge and skills, as compared to activities, such as massage or passive exercises. Therefore, sufficient explanation and education of the patients and their families are essential. In addition, it should be taken into consideration that involving highly distressed family members in rehabilitation may further worsen their own psychological state [152]. Furthermore, some reports have suggested the limited effectiveness of family participation in alleviating depression and anxiety symptoms in family members, indicating its limited impact on their mental health [157].

While the involvement of family members in the rehabilitation of critically ill patients has not been verified sufficiently, it is anticipated that future research will shed light on its effectiveness.

### Supplementary Information

**Additional file 1.** Financial and academic COIs and roles of committee members.

**Additional file 2.** Supplementary materials associated with the clinical practice guidelines.

## Abbreviations

6MWD: 6-Minute walk distance
ADL: Activity of daily living
BQ: Background question
CPG: Clinical practice guideline
CQ: Clinical question
DI: Disagreement index
EtD: Evidence-to-decision
FQ: Foreground question
GRADE: Grading of recommendations, assessment, development, and evaluation
GDG: Guideline Development Group
HRQOL: Health-related quality of life
JSICM: Japanese Society of Intensive Care Medicine
MD: Mean difference
MRC-SS: Medical Research Council sum score
QOL: Quality of life
RCT: Randomized controlled trial
SR: Systematic review
WG: Working group
