# Supplementary material for: Japanese Clinical Practice Guidelines for Rehabilitation in Critically Ill Patients 2023 (J-ReCIP 2023)
Source: J Intensive Care. 2023 Nov 7;11:47. doi: 10.1186/s40560-023-00697-w (PMC10629099; doi:10.1186/s40560-023-00697-w)
Supplement: Supplementary file 1 — Additional file 1. Financial and academic COIs and roles of committee members. [file 40560_2023_697_MOESM1_ESM.pdf]

Additional file

Disclosure of intellectual and financial conflicts of interest for each person who participated in the creation of this guideline.

Preparation role of each person who participated in the preparation of this guideline.

Committee Members and Directors in Charge

Name	Role of this Committee	Areas of Responsibility
Takeshi Unoki	Committee Chair	
Shunsuke Taito	Vice Chair<br>Member of Academic Guideline Promotion Group	WG7 group leader, WG1
Yusuke Kawai	Vice Chair<br>Member of Academic Guideline Promotion Group	WG2 group leader, WG8
Kei Hayashida	Committee Member<br>Head of Academic Guideline Promotion Group	
Fumihito Kasai	Committee Member	WG3 vice squad leader
Hideaki Sakuramoto	Committee Member	WG8 group leader
Kensuke Nakamura	Committee Member	WG5 group leader
Masakazu Saitoh	Committee Member	WG4 group leader
Morihide Ando	Committee Member	WG1 vice squad leader
Nobuyuki Sasaki	Committee Member	WG1 vice squad leader
Ryo Kozu	Committee Member	WG1 group leader
Ryuichi Saura	Committee Member	WG1 vice squad leader
Tatsuya Kawasaki	Committee Member	WG6 group leader
Yuki Iida	Committee Member	WG3 group leader
Yutaka Kondo	Committee Member<br>Member of Academic Guideline Promotion Group	W2, W5
Tetsuya Takahashi	Director in Charge	
Osamu Nishida	Advisor	

Academic Guideline Promotion Group

Name	Role of this Committee	Areas of Responsibility
Akira Ouchi	Member	WG1, WG3
Masatsugu Okamura	Member	WG4, WG8
Norimasa Yamamoto	Member	WG1, WG5
Saiko Okamoto	Member	
Shodai Yoshihiro	Member	WG2, WG7
Tomoki Kuribara	Member	WG4
Taisuke Yasaka	Member	WG3, WG6
Yujiro Matsuishi	Member	WG6

Working Group Members, Systematic Review Members

Name	Role of this Committee	Areas of Responsibility
Akira Kuriyama	Systematic Review Member<br>Member of Academic Guideline Promotion Group	WG3
Akiko Makino	Working Group Member	WG8
Asuka Tsuchiya	Working Group Member<br>Systematic Review Member	WG1
Chihiro Narita	Working Group Member<br>Systematic Review Member	WG2
Chihiro Takayama	Working Group Member	WG4
Daisuke Okada	Working Group Member<br>Systematic Review Member	WG1
Hiroshi Yonekura	Systematic Review Member	WG5
Hirotaka Masaki	Working Group Member<br>Systematic Review Member	WG1
Hiroyasu Inoue	Systematic Review Member	WG3
Junko Tatsuno	Working Group Member	WG5

	Systematic Review Member	
Kazuki Okura	Working Group Member<br>Systematic Review Member	WG2
Keibun Liu	Working Group Member<br>Systematic Review Member	WG5
Kengo Obata	Working Group Member	WG3
Kohei Ota	Working Group Member	WG8
Kota Yamauchi	Working Group Member<br>Systematic Review Member	WG7
Kotaro Hirakawa	Working Group Member	WG4
Masahiro Takahashi	Working Group Member<br>Systematic Review Member	WG1
Masanari Kuwabara	Working Group Member	WG5
Masaru Moromizato	Working Group Member	WG7
Masatoshi Hanada	Working Group Member	WG4
Mio Kitayama	Working Group Member<br>Systematic Review Member	WG2
Mitsuhiro Tamoto	Working Group Member	WG4
Naoki Kaneda	Working Group Member<br>Systematic Review Member	WG6
Naoki Sasanuma	Working Group Member<br>Systematic Review Member	WG1
Naonori Tashiro	Working Group Member<br>Systematic Review Member	WG1
Naoya Yanagi	Working Group Member	WG4
Natsuhiro Yamamoto	Working Group Member<br>Systematic Review Member	WG6
Nobuto Nakanishi	Systematic Review Member	WG5
Ryo Abe	Working Group Member	WG8
Ryosuke Matsuki	Working Group Member	WG4
Ryuichi Kusaba	Working Group Member<br>Systematic Review Member	WG6
Saori Miyagishima	Working Group Member<br>Systematic Review Member	WG2
Satoshi Endo	Working Group Member<br>Systematic Review Member	WG3, 7
Shinichi Watanabe	Systematic Review Member	WG3
Shinya Matsushima	Working Group Member<br>Systematic Review Member	WG5
Shunsuke Kina	Working Group Member	WG7
Tadashi Nagato	Working Group Member	WG5
Tadayoshi Nonoyama	Working Group Member<br>Systematic Review Member	WG2
Takahiko Tamura	Working Group Member<br>Systematic Review Member	WG5
Takahisa Otsuka	Working Group Member<br>Systematic Review Member	WG6
Takahito Iitsuka	Working Group Member<br>Systematic Review Member	WG6
Tomohiro Nawa	Working Group Member<br>Systematic Review Member	WG6
Wataru Matsuda	Working Group Member<br>Systematic Review Member	WG3
Yuhei Yamashita	Working Group Member<br>Systematic Review Member	WG3
Yuki Uchiyama	Working Group Member	WG1
Yuki Wakabayashi	Working Group Member<br>Systematic Review Member	WG1
Yukiko Katayama	Working Group Member<br>Systematic Review Member	WG3

Yusuke Tsutsumi	Working Group Member<br>Systematic Review Member	WG7

The policy for disclosure of conflicts of interest (COI) in this clinical practice guideline
Financial conflicts of interest (COI) and intellectual COI are disclosed in this appendix file.

COI type
This clinical practice guideline classifies COI into financial COI and intellectual (academic) COI and classifies them into Primary COI and Secondary COI based on their importance. Members with primary COI are not allowed to participate in discussions or voting on the recommendations, and secondary COI is only for disclosure (no restriction). Further, the following criteria for financial COI and intellectual COI are applied. Financial interest is payments received in exchange for services or any financial value derived from holdings. Intellectual interest is attachment to a certain academic ideas or activities that create the potential for cognitive biases, or attachment to a specific viewpoint.

Category	Financial COI	Intellectual COI	Restriction
Primary COI	Active relationship within 3 years met the following criteria<br>Committee chair ：Classification①<br>Committee members ：Classification②	Authorship of original studies, directly bearing on a recommendation<br>Peer-reviewed grant funding, directly bearing on a recommendation.	Restriction from all discussion and voting about recommendation
Secondary COI	Inactive relationship that meets the following criteria but have already expired (e.g., contracts have been terminated)	Other than the above	No restrictions

Financial COI
The following items are to be disclosed in accordance with criteria A, B and C below. Disclose retrospectively for the past three years from January 2019 to December 2021 (while those with voting rights extend from January 2020 to December 2022.).

Peer-reviewed grant funding from pharmaceutical manufacturers, etc. are also subject to COI. In cases where the person is in a position such as chief professor or department director, if the fund is obtained as for the entire department, they should be disclosed as COI (*see item C below).

A. Declarant's own declaration
Regarding the COI declaration of an individual researcher, disclosure must be made for the following items A-1 to A-9.

A-1
When the compensation for a leadership position and/or an advisory role in one private company, corporate body, or for-profit organization (hereinafter referred to as “private company, corporate body, or organization”) involved in medical research is one million yen or more per year.

A-2.
When the profit from stocks (total dividend and gain-on-sale) in one private company is one million yen or more per year, or when the ratio of stocks held is 5% or more of all shares in one private company.

A-3
When patent royalty and/or licensing fees for one patent and/or license from one private company, corporate body, or organization are one million yen or more per year.

A-4
When the daily allowance or honoraria (e.g. lecture fees) paid as compensation for the hours detained or labor for attending (or presenting at or providing advice at) meetings by one private company, corporate body, or organization is five hundred thousand yen or more per year.

A-5
When the total manuscript fee for writing brochures, articles on discussions, or other publications paid by one private company, corporate body, or organization is five hundred thousand yen or more per year.

A-6
When the total contract-based research funding (including funding for collaborative research, funded research, and drug trials) for medical research that the disclosing party may substantially make a decision on how to use paid by one private company, corporate body, or organization is one million yen or more per year.

A-7
When the total of contributions for scholarships provided by one private company, corporate body, or organization to a disclosing party, his/her course/class, or laboratory that the disclosing party may substantially make a decision on how to use is one million yen or more per year.

A-8
When a presenter belongs to a study group sponsored by a private company, corporate body, or organization; provided, however, that when the total contributions that the disclosing party may substantially make a decision on how to use is one million yen or more per year.

A-9
When the total amount paid by one private company, corporate body, or organization for travel, gifts or anything directly unrelated to research is fifty thousand yen or more per year.

【B. Spouses, first-degree relatives, and anyone who shares the income and assets of the people】

If spouses, first-degree relatives, and anyone who shares the income and assets of the people of a member met the following criteria, it should be indicated.

B-1
When the compensation for a leadership position and/or an advisory role in one private company, corporate body, or for-profit organization (hereinafter referred to as "private company, corporate body, or organization") involved in medical research is one million yen or more per year.

B-2.
When the profit from stocks (total dividend and gain-on-sale) in one private company is one million yen or more per year, or when the ratio of stocks held is 5% or more of all shares in one private company.

B-3
When patent royalty and/or licensing fees for one patent and/or license from one private company, corporate body, or organization are one million yen or more per year.

【C. Institutional COI】

When the disclosing party is currently or was in the past in a relationship of joint researcher or co-researcher with the chief executive of the research institute to which the disclosing party belongs or the chief of the department of such institute (university, hospital, faculty, or center), the COI must be disclosed in accordance with the designated form (Form 3-C) for the following matters. Regarding the amounts that require COI disclosure, criteria have been set for each item requiring disclosure as follows:

C-1
When the total contract based research funding (including funding for collaborative research, funded research, and drug trials) for medical research that the disclosing party may substantially make a decision on how to use paid by one private company, corporate body, or organization is ten million yen or more per year.

C-2
When the total contributions provided by one private company, corporate body, or organization to a disclosing party, the affiliate institute or department to which the disclosing party belongs or chief of such institute or department for which the disclosing party may substantially make a decision on how to use is two million yen or more per year

C-3
When there are any shares owned by the research institute or department to which the disclosing party belongs or the head thereof (in a joint researcher or co-researcher relationship within the past three years), patent royalties, or investment in a venture company, include such as institutional COI.

Intellectual COI

The following items are to be disclosed in accordance with criteria A, B and C below. The items in C-1 are treated as Primary COI. For items C-3 and C-4, if the research is based on acquired research funds, both the source of research funds and the name of the research should be disclosed and treated as a Primary COI.

A. Positions in academic organizations

If a member has a position of board member in a national academic organizations or equivalents including societies in all areas within 3 years (between January 1, 2019 and December 2021 while those with voting rights extend from January 2020 to December 2022.), the position and society name should be listed.

B. Involvement in the clinical guidelines

B-1

If a member has been involved in any medical guideline or its equivalent between January 2019, and December 2021 (while those with voting rights extend from January 2020 to December 2022), the name and position of the guideline should be indicated.

B-2

The member of a panel committee in the previous edition of the ARDS Clinical Practice Guidelines 2016, it should be indicated.

C. Involvement in related research

C-1

If a member is an author (co-author) of a paper that is included as primary original study in a systematic review for developing recommendation, the name of the paper and journal should be indicated. (It includes the period before the last three years.)

C-2

If a member is an author (or coauthor) of systematic review for developing recommendation, the name of the paper and the journal should be disclosed. (It includes the period before the last three years.)

C-3

If a member is an author of the study or speaker (including co-author or co-speaker) in a conference presentation related to clinical question in this guideline published or held between January 2019 and December 2021(while those with voting rights extend from January 2020 to December 2022), the name of the article, journal or conference should be indicated.

C-4

If a member is involved in the research related to the clinical questions in this guideline performed between January 2019 and December 2021(while those with voting rights extend from January 2020 to December 2022), the name of the research should be disclosed.

COI List (Financial COI）

Name	A-1	A-2	A-3	A-4	A-5	A-6	A-7	A-8	A-9	B-1	B-2	B-3	C-1	C-2	C-3
Takeshi Unoki	NA	NA	NA	NA	NA	NA	NA	NA	NA	NA	NA	NA	NA	NA	NA
Shunsuke Taito	NA	NA	NA	NA	NA	NA	NA	NA	NA	NA	NA	NA	NA	NA	NA
Yusuke Kawai	NA	NA	NA	NA	NA	NA	NA	NA	NA	NA	NA	NA	NA	NA	NA
Kei Hayashida	NA	NA	NA	NA	NA	NA	NA	NA	NA	NA	NA	NA	*1	NA	NA
Fumihito Kasai	NA	NA	NA	NA	NA	NA	NA	NA	NA	NA	NA	NA	NA	NA	NA
Hideaki Sakuramoto	NA	NA	NA	NA	NA	NA	NA	*2	NA	NA	NA	NA	NA	NA	NA
Kensuke Nakamura	NA	NA	NA	NA	NA	NA	NA	NA	NA	NA	NA	NA	NA	NA	NA
Masakazu Saitoh	NA	NA	NA	NA	NA	NA	NA	NA	NA	NA	NA	NA	NA	NA	NA
Morihide Ando	NA	NA	NA	NA	NA	NA	NA	NA	NA	NA	NA	NA	NA	NA	NA
Nobuyuki Sasaki	NA	NA	NA	*3	NA	NA	NA	NA	NA	NA	NA	NA	NA	NA	NA
Ryo Kozu	NA	NA	NA	*4	NA	NA	*5	NA	NA	NA	NA	NA	NA	NA	NA
Ryuichi Saura	NA	NA	NA	NA	NA	NA	NA	NA	NA	NA	NA	NA	NA	NA	NA
Tatsuya Kawasaki	NA	NA	NA	NA	NA	NA	NA	NA	NA	NA	NA	NA	NA	NA	NA
Yuki Iida	NA	NA	NA	NA	NA	NA	NA	NA	NA	NA	NA	NA	NA	NA	NA
Yutaka Kondo	NA	NA	NA	NA	NA	NA	NA	*6	NA	NA	NA	NA	NA	NA	NA
Tetsuya Takahashi	NA	NA	NA	NA	NA	NA	NA	*7	NA	NA	NA	NA	*8	NA	NA
Osamu Nishida	NA	NA	NA	NA	NA	*9	*10	NA	NA	NA	NA	NA	NA	NA	NA
Akira Ouchi	NA	NA	NA	NA	NA	NA	NA	NA	NA	NA	NA	NA	NA	NA	NA
Masatsugu Okamura	NA	NA	NA	NA	NA	NA	NA	NA	NA	NA	NA	NA	NA	NA	NA
Norimasa Yamamoto	NA	NA	NA	NA	NA	NA	NA	NA	NA	NA	NA	NA	NA	NA	NA
Saiko Okamoto	NA	NA	NA	NA	NA	NA	NA	NA	NA	NA	NA	NA	NA	NA	NA
Shodai Yoshihiro	NA	NA	NA	NA	NA	NA	NA	NA	NA	NA	NA	NA	NA	NA	NA
Tomoki Kuribara	NA	NA	NA	NA	NA	NA	NA	NA	NA	NA	NA	NA	NA	NA	NA
Taisuke Yasaka	NA	NA	NA	NA	NA	NA	NA	NA	NA	NA	NA	NA	NA	NA	NA
Yujiro Matsuishi	NA	NA	NA	NA	NA	NA	NA	NA	NA	NA	NA	NA	NA	NA	NA
Akiko Makino	NA	NA	NA	NA	NA	NA	NA	NA	NA	NA	NA	NA	NA	NA	NA
Akira Kuriyama	NA	NA	NA	NA	NA	NA	NA	NA	NA	NA	NA	NA	NA	NA	NA
Asuka Tsuchiya	NA	NA	NA	NA	NA	NA	NA	NA	NA	NA	NA	NA	NA	NA	NA
Chihiro Narita	NA	NA	NA	NA	NA	NA	NA	NA	NA	NA	NA	NA	NA	NA	NA
Chihiro Takayama	NA	NA	NA	NA	NA	NA	NA	NA	NA	NA	NA	NA	NA	NA	NA
Daisuke Okada	NA	NA	NA	NA	NA	NA	NA	NA	NA	NA	NA	NA	NA	NA	NA
Hiroshi Yonekura	NA	NA	NA	NA	NA	NA	NA	NA	NA	NA	NA	NA	NA	NA	NA
Hirotaka Masaki	NA	NA	NA	NA	NA	NA	NA	NA	NA	NA	NA	NA	NA	NA	NA
Hiroyasu, Inoue	NA	NA	NA	NA	NA	NA	NA	NA	NA	NA	NA	NA	NA	NA	NA
Junko Tatsuno	NA	NA	NA	NA	NA	NA	NA	NA	NA	NA	NA	NA	NA	NA	NA

Kazuki Okura	NA	NA	NA	NA	NA	NA	NA	NA	NA	NA	NA	NA	NA	NA	NA
Keibun Liu	*11	NA	NA	NA	NA	NA	NA	NA	NA	NA	NA	NA	NA	NA	NA
Kengo Obata	NA	NA	NA	NA	NA	NA	NA	NA	NA	NA	NA	NA	NA	NA	NA
Kohei Ota	NA	NA	NA	NA	NA	NA	*12	NA	NA	NA	NA	NA	NA	NA	NA
Kota Yamauchi	NA	NA	NA	NA	NA	NA	NA	NA	NA	NA	NA	NA	NA	NA	NA
Kotaro Hirakawa	NA	NA	NA	NA	NA	NA	NA	NA	NA	NA	NA	NA	NA	NA	NA
Masahiro Takahashi	NA	NA	NA	NA	NA	NA	NA	NA	NA	NA	NA	NA	NA	NA	NA
Masanari Kuwabara	NA	NA	NA	NA	NA	NA	NA	NA	NA	NA	NA	NA	NA	NA	NA
Masaru Moromizato	NA	NA	NA	NA	NA	NA	NA	NA	NA	NA	NA	NA	NA	NA	NA
Masatoshi Hanada	NA	NA	NA	NA	NA	NA	NA	NA	NA	NA	NA	NA	NA	NA	NA
Mio Kitayama	NA	NA	NA	NA	NA	NA	NA	NA	NA	NA	NA	NA	NA	NA	NA
Mitsuhiro Tamoto	NA	NA	NA	NA	NA	NA	NA	NA	NA	NA	NA	NA	NA	NA	NA
Naoki Kaneda	NA	NA	NA	NA	NA	NA	NA	NA	NA	NA	NA	NA	NA	NA	NA
Naoki Sasanuma	NA	NA	NA	NA	NA	NA	NA	NA	NA	NA	NA	NA	NA	NA	NA
Naonori Tashiro	NA	NA	NA	NA	NA	NA	NA	NA	NA	NA	NA	NA	NA	NA	NA
Naoya Yanagi	NA	NA	NA	NA	NA	NA	NA	NA	NA	NA	NA	NA	NA	NA	NA
Natsuhiro Yamamoto	NA	NA	NA	NA	NA	NA	NA	NA	NA	NA	NA	NA	NA	NA	NA
Nobuto Nakanishi	NA	NA	NA	NA	NA	NA	NA	NA	NA	NA	NA	NA	NA	NA	NA
Ryosuke Matsuki	NA	NA	NA	NA	NA	NA	NA	NA	NA	NA	NA	NA	NA	NA	NA
Ryo Abe	NA	NA	NA	NA	NA	NA	NA	NA	NA	NA	NA	NA	NA	NA	NA
Ryuichi Kusaba	NA	NA	NA	NA	NA	NA	NA	NA	NA	NA	NA	NA	NA	NA	NA
Saori Miyagishima	NA	NA	NA	NA	NA	NA	NA	NA	NA	NA	NA	NA	NA	NA	NA
Satoshi Endo	NA	NA	NA	NA	NA	NA	NA	NA	NA	NA	NA	NA	NA	NA	NA
Shinichi Watanabe	NA	NA	NA	NA	NA	NA	NA	NA	NA	NA	NA	NA	NA	NA	NA
Shinya Matsushima	NA	NA	NA	NA	NA	NA	NA	NA	NA	NA	NA	NA	NA	NA	NA
Shunsuke Kina	NA	NA	NA	NA	NA	NA	NA	NA	NA	NA	NA	NA	NA	NA	NA
Tadashi Nagato	NA	NA	NA	NA	NA	NA	NA	NA	NA	NA	NA	NA	NA	NA	NA
Tadayoshi Nonoyama	NA	NA	NA	NA	NA	NA	NA	NA	NA	NA	NA	NA	NA	NA	NA
Takahiko Tamura	NA	NA	NA	NA	NA	NA	NA	NA	NA	NA	NA	NA	NA	NA	NA
Takahisa Otsuka	NA	NA	NA	NA	NA	NA	NA	NA	NA	NA	NA	NA	NA	NA	NA
Takahito Iitsuka	NA	NA	NA	NA	NA	NA	NA	NA	NA	NA	NA	NA	NA	NA	NA
Tomohiro Nawa	NA	NA	NA	NA	NA	NA	NA	NA	NA	NA	NA	NA	NA	NA	NA
Wataru Matsuda	NA	NA	NA	NA	NA	NA	NA	NA	NA	NA	NA	NA	NA	NA	NA
Yuhei Yamashita	NA	NA	NA	NA	NA	NA	NA	NA	NA	NA	NA	NA	NA	NA	NA
Yuki Uchiyama	NA	NA	NA	NA	NA	NA	NA	NA	NA	NA	NA	NA	NA	NA	NA
Yuki Wakabayashi	NA	NA	NA	NA	NA	NA	NA	NA	NA	NA	NA	NA	NA	NA	NA
Yukiko Katayama	NA	NA	NA	NA	NA	NA	NA	NA	NA	NA	NA	NA	NA	NA	NA
Yusuke Tsutsumi	NA	NA	NA	NA	NA	NA	NA	NA	NA	NA	NA	NA	NA	NA	NA

NA: Not applicable

＊1 TAIYOU NIPPON SANSO, 2021

＊2 New nursing Corporation, Endowed chair, 2022

＊3 Teijin Pharma, 2021, 2022

＊4 Teijin, 2019
Boehringer Ingelheim, 2022

＊5 Philips Japan, 2021

＊6 TOPPAN, 2021

＊7 Philips Japan, Toho Holdings, Inter Reha, Asahi KASEI
Department of Digital Medicine Juntendo University

＊8 Philips Japan, Collaborating research, 2022
Toho Holdings, Collaborating research, 2022
Kyocera・FUKUDA・Inter Reha・AMI, Collaborating research
MF Consulting, Collaborating research

＊9 Maruishi Pharmaceutical, Clinical trial, 2019,2020,2021
ONO PHARMACEUTUICAL, Clinical trial, 2019
Fortrea Japan (Labcorp Drug Development Japan), Clinical trial, 2021
Healio, Clinical trial, 2021

＊10 Baxter, 2019
SHIONOGI, 2019, 2020
ONO Pharmaceutical, 2019
Asahi KASEI, 2020
Asahi KASEI Pharma, 2021, 2022

＊11 Senko Medical Instrument, Consultant, 2020

＊12 HIROSHIMA ROTARY CLUB, 2022

COI List（Intellectual COI） (Items without names in English are listed in Japanese.)

Name	A : Society, Role	B-1 : Guideline, Role	B-2: Member of previous version	C-1: Title, Journal	C-2: Title, Journal, C-3: Title, Conference	C-4: Related research
Takeshi Unoki	The Japanese Society of Intensive Care Medicine, Director	J-SSCG2020, WG member	A	NA	＊18	NA
Shunsuke Taito	NA	J-SSCG2020, AC member/理学療法ガイドライン第2, SR member/ARDS clinical practice guideline 2021, 領域責任委員/ J-SSCG2024, Committee Member	NA	NA	＊2	NA
Yusuke Kawai	Japanese Society for Early Mobilization, director	J-SSCG2020, AC member WG member SR member/J-SSCG2024, Committee Member /気管吸引ガイドライン2023〔改訂第 3 版〕, Committee Member	NA	NA	NA	＊7, 15
Kei Hayashida	NA	J-SSCG2020, AC member/Japanese Resuscitation Council Resuscitation Care Guidelines 2020, WG member	NA	NA	NA	NA
Fumihito Kasai	NA	NA	NA	NA	＊3, 4	＊16
Hideaki Sakuramoto	NA	J-SSCG2020, WG member	Y	NA	NA	NA
Kensuke Nakamura	Japanese Society for Early Mobilization, director<br>Japan Society for Homecare and Emergency Medicine, director<br>Japanese Association for Acute Medicine, director	J-SSCG2020, WG member/ 重症患者の栄養療法ガイドライン 2024, Vice Chair	NA	＊1	＊1, 18	＊1
Masakazu Saitoh	Japanese Society of Renal Rehabilitation, director	呼吸療法ガイドライン第 2 版 SR member/心血管理学療法ガイドライン, SR member	Y	NA	NA	NA
Morihide Ando	Japanese Society for Respiratory Care and Rehabilitation, director	COPD 診断と治療のためのガイドライン 2022, SR Committee Member	Y	NA	NA	NA
Nobuyuki Sasaki	Japanese Association of Rehabilitation Medicine 事務局, executive director<br>The Japanese Society of Acute Rehabilitation Medicine, auditor	Japan Stroke Society guideline 2021, Committee Member	NA	NA	NA	NA
Ryo Kozu	The Japanese Society for Respiratory Care and Rehabilitation, director<br>Japanese Society for Respiratory Care Medicine 職種指定理事<br>Japanese Society of Physical Respiratory Therapy, director	ARDS clinical practice guideline 2021, パネル会議メンバー/理学療法ガイドライン第2版, Committee Member	Y	NA	＊5	NA
Ryuichi Saura	Japanese Association of Rehabilitation Medicine, director<br>The Japanese Society of Acute Rehabilitation Medicine, director<br>The Japanese Organization for Education and Certification of Rehabilitation Medicine, director<br>Japan Stroke Association, director<br>Japan disaster Rehabilitation Assistance Team 副代表<br>Duskin AINOWA Foundation, board member<br>Japanese Association for Rheumatism Rehabilitation 代表世話人<br>日本がんリハビリテーション研究会, director	Guidelines for Cancer Rehabilitation（第2版）, Committee Member/メディカルスタッフのためのライフステージに応じた関節リウマチ患者支援ガイド, Research member	NA	NA	NA	NA

Tatsuya Kawasaki	NA	J-SSCG2020, Committee Member/ J-SSCG2024, Committee Member / ARDS clinical practice guideline 2021, Pediatric Panel Members/Japanese Resuscitation Council Resuscitation Care Guidelines 2020, 作業部会メンバー/Japanese rapid/living recommendations on drug management for COVID-19, Advisory board member/COVID-19 関連多系統炎症性症候群 (MIS-C/PIMS) 診療コンセンサスステートメント, WG member/日本小児科学会予防接種・感染症対策委員会および日本集中治療医会小児集中治療委員会日本小児集中治療連絡協議会 COVID-19 ワーキンググループ 活動報告第2報, WG member	NA	NA	NA	NA
Yuki Iida	Japanese Society of Cardiovascular Physical Therapy, director<br>The Japanese Society for Respiratory Care and Rehabilitation, director	J-SSCG2020, WG member	Y	NA	NA	NA
Yutaka Kondo	NA	J-SSCG2020, WG member/ J-SSCG2024, Committee Member/ARDS clinical practice guideline 202, 統括本部委員/Japanese Resuscitation Council Resuscitation Care Guidelines 2020, Committee Member/JCS 2022 Guideline Perioperative Cardiovascular Assessment and Management for Non-cardiac Surgery , 協力委員/熱中症診療ガイドライン 2024, 副編集長	NA	NA	＊18	NA
Tetsuya Takahashi	Japanese Association of Cardiac Rehabilitation, vice director<br>Japanese Society of Cardiovascular Physical Therapy, vice director<br>Japanese Physical Association, vice director	JCS/JACR 2021 Guideline on Rehabilitation in Patients with Cardiovascular Disease, 班員/理学療法ガイドライン第2版, 循環器班・副班長	Y	NA	NA	NA
Osamu Nishida	The Japanese Society of Intensive Care Medicine, President<br>Japanese Society of Blood Purification in Critical Care, President<br>Japanese Society of Respiratory Care Medicine, director<br>The Japan Shock Society, director<br>The Japanese Society for Therapeutics and Engineering, director<br>Japanese Society for Clinical Monitoring, director	J-SSCG2020, Director in Charge/ Japanese guidelines for the management of Pain, Agitation, and Delirium in intensive care unit (J-PAD), Director in Charge/集中治療を要する重症患者の広域搬送ガイドライ, President/遠隔 ICU 設置と運用に関するガイドライン改訂版 2023 年 5 月, President/日本集中治療医学会集中治療に係るタスク・シフト/フェアに関する安全管理指, President/臓器提供を見据えた患者評価・管理と術中管理のためのマニュアル(厚生労働科学特別研究事業), Research member/Japanese rapid/living recommendations on drug management for COVID-19, director in charge/ARDS clinical practice guideline 2021, President/集中治療部設置のための指針2022年改訂版, President/集中治療部設置のための指針2021 年改訂について 空調設備に関する改訂方針と提言, president/ 日本集中治療医学会 集中治療室における安全管理指針, President/新型コロナウィルス感染症流行に際しての医療資源配分の観点から治療の差し控え・中止についての提言, President/集中治療室における薬剤師の活動方針, President	Y	NA	NA	NA
Akira Kuriyama	NA	NA	NA	NA	NA	NA
Akira Ouchi	NA	J-SSCG2020, WG member	NA	NA	NA	NA
Masatsugu Okamura	NA	NA	NA	NA	NA	NA

Norimasa Yamamoto	NA	NA	NA	NA	＊18	NA
Saiko Okamoto	NA	NA	NA	NA	NA	NA
Shodai Yoshihiro	NA	J-SSCG2020, WG member/ARDS clinical practice guideline 2021, SR member	NA	NA	NA	NA
Tomoki Kuribara	NA	NA	NA	NA	NA	NA
Taisuke Yasaka	NA	NA	NA	NA	＊6	NA
Yujiro Matsuishi	NA	NA	NA	NA	NA	NA
Akiko Makino	NA	NA	NA	NA	NA	NA
Akira Kuriyama	NA	NA	NA	NA	NA	NA
Asuka Tsuchiya	NA	J-SSCG2020 WG member, SR member/急性膵炎診療ガイドライン 2021, Committee Member/血管塞栓術に用いるゼラチンスポンジガイドライン, Committee Member	NA	NA	NA	NA
Chihiro Narita	NA	J-SSCG2020, SR member/ARDS clinical practice guideline 2021, サポート班/領域責任委員 SR 責任者/J-SSCG2020 COVID-19living guideline, WG メンバー/ J-SSCG2024, AC member	NA	NA	NA	NA
Chihiro Takayama	NA	NA	NA	NA	NA	NA
Daisuke Okada	NA	理学療法ガイドライン第 2 版, SR member	NA	NA	NA	NA
Hiroshi Yonekura	NA	J-SSCG2020, WG member/ARDS clinical practice guideline 202, Committee Member/周術期禁煙ガイドライン改定	NA	NA	＊18	NA
Hirotaka Masaki	NA	NA	NA	NA	NA	NA
Hiroyasu Inoue	NA	理学療法ガイドライン第 2 版, SR member	NA	NA	＊8	＊17
Junko Tatsuno	NA	NA	NA	NA	＊18	NA
Kazuki Okura	NA	理学療法ガイドライン第 2 版, SR member	NA	NA	NA	NA
Keibun Liu	NA	NA	NA	NA	＊7	NA
Kengo Obata	NA	NA	NA	NA	NA	NA
Kohei Ota	NA	J-SSCG2020, WG member/ARDS clinical practice guideline 2021, SR member	NA	NA	NA	NA
Kota Yamauchi	NA	日本理学療法士協会・理学療法ガイドライン, SR member/ARDS clinical practice guideline 2021, SR member	NA	NA	NA	NA
Kotaro Hirakawa	NA	NA	NA	NA	NA	NA
Masahiro Takahashi	NA	理学療法ガイドライン第 2 版, SR member	Y	NA	NA	NA
Masanari Kuwabara	NA	新型コロナウイルス感染症診療の手引き別冊 執筆協力者/循環器病対策推進基本計画 厚生労働省（事務局）	NA	NA	NA	NA
Masaru Moromizato	NA	NA	NA	NA	NA	NA

Masatoshi Hanada	NA	理学療法ガイドライン 第2班 COPD 班, 副班長	NA	NA	NA	NA
Mio Kitayama	NA	NA	NA	NA	NA	NA
Mitsuhiro Tamoto	NA	NA	NA	NA	NA	NA
Naoki Kaneda	NA	理学療法ガイドライン第 2 版, SR member	NA	NA	NA	NA
Naoki Sasanuma	NA	理学療法ガイドライン第 2 版, SR member	Y	NA	NA	NA
Naonori Tashiro	NA	NA	Y	NA	＊9	NA
Naoya Yanagi	NA	NA	NA	NA	NA	NA
Natsuhiro Yamamoto	NA	NA	NA	NA	NA	NA
Nobuto Nakanishi	NA	NA	NA	NA	＊10, 18	＊10
Ryo Abe	NA	理学療法ガイドライン第 2 版, SR member	NA	NA	NA	NA
Ryosuke Matsuki	有	理学療法ガイドライン（第2版）, SR member	NA	NA	＊11	NA
Ryuichi Kusaba	NA	NA	NA	NA	NA	NA
Saori Miyagishima	NA	NA	NA	NA	NA	NA
Satoshi Endo	NA	ICU における COVID-19 患者に対するリハビリテーション Q&A, WG member	NA	NA	NA	NA
Shinichi Watanabe	NA	NA	NA	NA	NA	NA
Shinya Matsushima	有	理学療法ガイドライン（第2版）, SR member	NA	NA	＊12, 18	NA
Shunsuke Kina	NA	NA	NA	NA	NA	NA
Tadashi Nagato	NA	J-SSCG2020, SR member/ARDS clinical practice guideline 2021, SR member /結核診療ガイドライン, SR member/Japan Stroke Society guideline 2021, task force member	NA	NA	NA	NA
Tadayoshi Nonoyama	NA	NA	NA	NA	NA	NA
Takahiko Tamura	NA	NA	NA	NA	＊13, 18	NA
Takahisa Otsuka	NA	NA	NA	NA	NA	NA
Takahito Iitsuka	NA	理学療法ガイドライン第 2 版, SR member	NA	NA	＊14	NA
Tomohiro Nawa	NA	NA	NA	NA	NA	NA
Wataru Matsuda	NA	NA	NA	NA	NA	NA
Yuhei Yamashita	NA	NA	NA	NA	NA	NA
Yuki Uchiyama	NA	NA	NA	NA	NA	NA

Yuki Wakabayashi	NA	NA	NA	NA	NA	NA
Yukiko Katayama	NA	NA	NA	NA	NA	NA
Yusuke Tsutsumi	NA	J-SSCG2020, WG member	NA	NA	NA	NA

(Abbreviation)
J-SSCG2020: Japanese Surviving Sepsis Clinical Guideline 2020, NA: Not applicable, SR: Systematic review, WG: Working group

<Detail of Intellectual COI>

C-1

＊1

Nakamura K, Kihata A, Naraba H, et al. Efficacy of belt electrode skeletal muscle electrical stimulation on reducing the rate of muscle volume loss in critically ill patients: A randomized controlled trial *J Rehabil Med*. 2019;51(9):705-711.

Nakamura K, Nakano H, Naraba H, et al. High protein versus medium protein delivery under equal total energy delivery in critical care: A randomized controlled trial. *Clin Nutr*. 2021;40(3):796-803.

Nakamura K, Kihata A, Naraba H, et al. β-Hydroxy-β-methylbutyrate, Arginine, and Glutamine Complex on Muscle Volume Loss in Critically Ill Patients: A Randomized Control Trial. *JPEN J Parenter Enteral Nutr*. 2020;44(2):205-212.

C-2 C-3

＊2

Taito S, Yamauchi K, Tsujimoto Y, Banno M, Tsujimoto H, Kataoka Y. Does enhanced physical rehabilitation following intensive care unit discharge improve outcomes in patients who received mechanical ventilation? A systematic review and meta-analysis. *BMJ Open*. 2019;9(6):e026075.

＊3

The Japanese Situation of Swallowing Evaluation & Treatment, 2th World Dysphagia, 2021

＊4

本邦の集中治療室における摂食嚥下診療調査, 日本摂食嚥下リハビリテーション学会雑誌, 2022

＊5

ICU における早期リハビリテーションの取り組み：理学療法士の立場から ICU 多職種合同シンポジウム「ICU における早期リハビリテーションを考える」 第 4 回日本集中治療医学会九州支部学術集会, 2020

Watanabe S, Kotani T, Taito S, et al. Determinants of gait independence after mechanical ventilation in the intensive care unit: a Japanese multicenter retrospective exploratory cohort study. *J Intensive Care*. 2019;7:53.

＊6

集中治療室における早期リハビリテーション実践の実態：横断調査,日本クリティカルケア看護学会学術集会,2020.

本邦集中治療室における重症患者に対するリハビリテーション実践と患者及び組織特性の関連の検討：横断研究,日本集中治療医学会,2021.

ICU に入室する急性呼吸不全患者に対する早期離床：最新の知見に基づき臨床実践の課題を考える, 日本集中治療医学会, 2022.

＊7

集中治療室重症患者の予後に関する調査, 2021

＊8

携帯型超音波機器を用いた嚥下機能評価と多職種連携による経口摂取を再開できた 1 例,日本リハビリテーション看護学会, 2021

携帯型超音波機器によるベッドサイド嚥下評価の試み　梨状窩通過時の嚥下物検出の信頼性の検証, 日本摂食・嚥下リハビリテーション学会,2021

48 時間以上の経口挿管下人工呼吸管理患者における抜管後誤嚥性肺炎発症の予測因子の検証, 昭和学士会雑誌, 2019

＊9

48 時間以上の人工呼吸装着患者の退院時自立補講の関連因子, 日本集中治療医学会雑誌, 2021

48 時間以上経口挿管下人工呼吸管理患者における抜管後誤嚥生肺炎発症の予測因子の検証, 昭和学士会誌, 2019

＊10

Nakanishi N, Oto J, Tsutsumi R, et al. Effect of Electrical Muscle Stimulation on Upper and Lower Limb Muscles in Critically Ill Patients: A Two-Center Randomized Controlled Trial. *Crit Care Med*. 2020;48(11):e997-e1003.

＊11

Matsuki R, Kojima N, Watanabe K, et al. Impact of a Rehabilitation Protocol and a Dedicated Therapist in the Intensive Care Unit on Physical Function and Activities of Daily Living. *Prog Rehabil Med*. 2020;5:20200027.

＊12

Matsushima S, Yoshida M, Yokoyama H, et al. Effects on physical performance of high protein intake for

critically ill adult patients admitted to the intensive care unit: A retrospective propensity-matched analysis. *Nutrition*. 2021;91-92:111407.
ICU 入室 7 日目の蛋白投与量は転帰時の ADL に影響を与えるか？ 集中治療医学会, 2020
集中治療領域における N ST ガイドライン検証　重症患者における急性期にエネルギー投与量が ADL の転帰に与える影響　単施設後ろ向きコホート研究, 日本臨床栄養代謝学会学術集会, 2019
重症患者における急性期の蛋白投与量が早期離床・リハビリテーションの効果に与える影響, 集中治療医学会, 2019
＊13
Tamura T, Yatabe T, Yokoyama M, Nishimura M. Current status of studies about optimal composition of amino acid and protein for ICU patients: systematic review. *Anaesth Intensive Care*. 2014;42(6):806-807.
＊14
当院 PICU 入室患者の運動機能回復に与える因子の検討, 第 49 回日本集中治療医学会学術集会, 2022
当院 PICU における早期離床プロトコル導入の効果について－離床への効果について着目して－, 第 8 回小児理学療法学会, 2021
当院 PICU での離床と運動機能回復との関係, 第 47 回日本集中治療医学会学術集会, 2020
C-4
＊15
集中治療室に入室した重症患者とその家族の集中治療後症候群の疫学調査, 2022
＊16
Spronk PE, Spronk LEJ, Egerod I, et al. Dysphagia in Intensive Care Evaluation (DICE): An International Cross-Sectional Survey. *Dysphagia*. 2022;37(6):1451-1460.
＊17
携帯型エコーを用いた嚥下評価の可視化調査, 2022
携帯型超音波機器によるベッドサイド嚥下評価の検者間信頼性の検証, 2020
48 時間以上の経口挿管下人工呼吸管理患者における抜管後嚥下障害の調査, 2019
＊18
Nakanishi N, Matsushima S, Tatsuno J, et al. Impact of Energy and Protein Delivery to Critically Ill Patients: A Systematic Review and Meta-Analysis of Randomized Controlled Trials. *Nutrients*. 2022;14(22):4849.
