# Supplementary material for: Japanese Clinical Practice Guidelines for Rehabilitation in Critically Ill Patients 2023 (J-ReCIP 2023)
Source: J Intensive Care. 2023 Nov 7;11:47. doi: 10.1186/s40560-023-00697-w (PMC10629099; doi:10.1186/s40560-023-00697-w)
Supplement: Supplementary file 2 — Additional file 2. Supplementary materials associated with the clinical practice guidelines. [file 40560_2023_697_MOESM2_ESM.pdf]

CQ 1
P: Adult patient with critical illness
I: Protocolized rehabilitation in the intensive care unit
C: Usual care

| Certainty assessment | | | | | | | № of patients | | Effect | | Certainty | Importance |
|---|---|---|---|---|---|---|---|---|---|---|---|---|
| № of studies | Study design | Risk of bias | Inconsistency | Indirectness | Imprecision | Other considerations | Intervention | Comparison | Relative (95% CI) | Absolute (95% CI) | | |
| **Fundamental Motion (assessed with: PFIT, IMS, MMS, SOMS)** | | | | | | | | | | | | |
| 6 | randomised trials | serious [a] | serious [b] | not serious | serious [c] | none | 307 | 288 | - | SMD **0.62 higher** (0.01 higher to 1.23 higher) | ⊕◯◯◯ Very low | CRITICAL |
| **Activities of Daily Living (assessed with: FIM, BI, mmFIM, SPPB)** | | | | | | | | | | | | |
| 5 | randomised trials | very serious [d] | serious [e] | not serious | serious [f] | none | 314 | 327 | - | SMD **0.15 higher** (0.27 lower to 0.57 higher) | ⊕◯◯◯ Very low | CRITICAL |
| **Muscle Strength (assessed with: MRC-SS)** | | | | | | | | | | | | |
| 5 | randomised trials | very serious [d] | serious [g] | not serious | serious [f] | none | 137 | 135 | - | MD **4.52 higher** (1.54 lower to 10.59 higher) | ⊕◯◯◯ Very low | CRITICAL |
| **Duration of Mechanical Ventilation (days)** | | | | | | | | | | | | |
| 16 | randomised trials | very serious [d] | serious [h] | not serious | not serious | publication bias strongly suspected [i] | 584 | 581 | - | MD **1.28 day lower** (1.68 lower to 0.87 lower) | ⊕◯◯◯ Very low | CRITICAL |
| **Length of Stay in the ICU (days)** | | | | | | | | | | | | |
| 19 | randomised trials | very serious [d] | serious [j] | not serious | not serious | none | 925 | 913 | - | MD **1.53 day lower** (2.3 lower to 0.77 lower) | ⊕◯◯◯ Very low | CRITICAL |
| **Incidence of Delirium in the ICU** | | | | | | | | | | | | |
| **0** | | | | | | | | | | | - | CRITICAL |
| **Any Adverse Event** | | | | | | | | | | | | |
| 7 | randomised trials | serious [a] | serious [k] | not serious | serious [l] | none | 39/489 (8.0%) | 43/505 (8.5%) | **RR 0.72** (0.28 to 1.83) | **24 fewer per 1,000** (from 61 fewer to 71 more) | ⊕◯◯◯ Very low | CRITICAL |

**CI:** confidence interval; **MD:** mean difference; **RR:** risk ratio; **SMD:** standardised mean difference

## Explanations

a. Downgrade one level due to risk of bias (blinding of participants and personnel).
b. Downgrade one level due to considerable heterogeneity ($I^2$ = 92%).
c. Downgrade one level due to smaller than OIS.
d. Downgrade two levels due to risk of bias (blinding of participants and personnel and incomplete outcome data).
e. Downgrade one level due to considerable heterogeneity ($I^2$ = 85%).

f. Downgrade one level due to the 95% confidence interval overlaps 0 and smaller than OIS.
g. Downgrade one level due to considerable heterogeneity ($I^2$= 82%).
h. Downgrade one level due to considerable heterogeneity ($I^2$ = 93%).
i. Detection of serious publication bias from Egger test (P < .01) and asymmetry of funnel plot.
j. Downgrade one level due to considerable heterogeneity ($I^2$ = 89%).
k. Downgrade one level due to substantial heterogeneity ($I^2$= 65%).
l. Downgrade one level due to the 95% confidence interval overlaps 1 and smaller than OIS.

| | JUDGEMENT | | | | | | |
|---|---|---|---|---|---|---|---|
| PROBLEM | No | Probably no | Probably yes | Yes | | Varies | Don't know |
| DESIRABLE EFFECTS | Trivial | Small | Moderate | Large | | Varies | Don't know |
| UNDESIRABLE EFFECTS | Large | Moderate | Small | Trivial | | Varies | Don't know |
| CERTAINTY OF EVIDENCE | Very low | Low | Moderate | High | | | No included studies |
| VALUES | Important uncertainty or variability | Possibly important uncertainty or variability | Probably no important uncertainty or variability | No important uncertainty or variability | | | |
| BALANCE OF EFFECTS | Favors the comparison | Probably favors the comparison | Does not favor either the intervention or the comparison | Probably favors the intervention | Favors the intervention | Varies | Don't know |
| ACCEPTABILITY | No | Probably no | Probably yes | Yes | | Varies | Don't know |
| FEASIBILITY | No | Probably no | Probably yes | Yes | | Varies | Don't know |

CQ2
P: Adult patient with critical illness
I: At least two rehabilitation sessions per day in the ICU
C: Only one rehabilitation session per day in the ICU

| Certainty assessment | | | | | | | № of patients | | Effect | | Certainty | Importance |
|---|---|---|---|---|---|---|---|---|---|---|---|---|
| № of studies | Study design | Risk of bias | Inconsistency | Indirectness | Imprecision | Other considerations | Intervention | Comparison | Relative (95% CI) | Absolute (95% CI) | | |
| **Fundamental Motion (Modified Rivermead Mobility Index)** | | | | | | | | | | | | |
| 1 | randomised trials | very serious[a] | not serious | not serious | very serious[b] | none | 112 | 104 | - | MD **3 higher** (0.33 higher to 5.67 higher) | ⨁◯◯◯ Very low | CRITICAL |
| **Activities of Daily Living (FIM, Modified Barthel Index)** | | | | | | | | | | | | |
| 2 | randomised trials | serious[c] | not serious | not serious | very serious[d] | none | 109 | 95 | - | SMD **0.22 higher** (0.05 lower to 0.5 higher) | ⨁◯◯◯ Very low | CRITICAL |
| **Muscle Strength (MRC-SS)** | | | | | | | | | | | | |
| 2 | randomised trials | serious[c] | not serious | not serious | very serious[d] | none | 45 | 42 | - | MD **2.17 lower** (5.62 lower to 1.29 higher) | ⨁◯◯◯ Very low | CRITICAL |
| **Duration of Mechanical Ventilation** | | | | | | | | | | | | |
| 6 | randomised trials | very serious[e] | serious[f] | not serious | serious[g] | none | 147 | 144 | - | MD **2.26 day lower** (3.86 lower to 0.65 lower) | ⨁◯◯◯ Very low | CRITICAL |
| **Length of Stay in the ICU** | | | | | | | | | | | | |
| 7 | randomised trials | very serious[e] | not serious | not serious | serious[g] | none | 271 | 262 | - | MD **2.24 day lower** (4.02 lower to 0.46 lower) | ⨁◯◯◯ Very low | CRITICAL |
| **Incidence of Delirium in the ICU** | | | | | | | | | | | | |
| **0** | | | | | | | | | | | | |
| **Any Adverse Events** | | | | | | | | | | | | |
| 3 | randomised trials | serious[c] | not serious | not serious | serious[g] | none | 2/205 (1.0%) | 0/217 (0.0%) | **RR 3.08** (0.33 to 29.10) | **10 more per 1,000** (from 10 fewer to 20 more) | ⨁⨁◯◯ Low | CRITICAL |

**CI:** confidence interval; **MD:** mean difference; **RR:** risk ratio; **SMD:** standardised mean difference

## Explanations

a. Downgraded two levels due to risk of bias (blinding of participants and personnel, and blinding of outcome assessment).
b. Downgraded two levels due to a smaller sample size than OIS and a wide range of 95% confidence interval.
c. Downgraded one level due to risk of bias (blinding of participants and personnel).
d. Downgraded two levels due to a smaller sample size than OIS and a wide range of 95% confidence interval straddling 1.
e. Downgraded two levels due to risk of bias (allocation concealment, blinding of outcome assessment).
f. Downgraded one level due to substantial heterogeneity (I2 = 53%).
g. Downgraded one level due to a smaller sample size than OIS

| | JUDGEMENT | | | | | | |
|---|---|---|---|---|---|---|---|
| **PROBLEM** | No | Probably no | Probably yes | Yes | | Varies | Don't know |
| **DESIRABLE EFFECTS** | Trivial | Small | Moderate | Large | | Varies | Don't know |
| **UNDESIRABLE EFFECTS** | Large | Moderate | Small | Trivial | | Varies | Don't know |
| **CERTAINTY OF EVIDENCE** | Very low | Low | Moderate | High | | | No included studies |
| **VALUES** | Important uncertainty or variability | Possibly important uncertainty or variability | Probably no important uncertainty or variability | No important uncertainty or variability | | | |
| **BALANCE OF EFFECTS** | Favors the comparison | Probably favors the comparison | Does not favor either the intervention or the comparison | Probably favors the intervention | Favors the intervention | Varies | Don't know |
| **ACCEPTABILITY** | No | Probably no | Probably yes | Yes | | Varies | Don't know |
| **FEASIBILITY** | No | Probably no | Probably yes | Yes | | Varies | Don't know |

CQ 3-1

P: Patient with critical illness

I: Neuromuscular electrical stimulation

C: Non-neuromuscular electrical stimulation

| Certainty assessment | | | | | | | № of patients | | Effect | | Certainty | Importance |
|---|---|---|---|---|---|---|---|---|---|---|---|---|
| № of studies | Study design | Risk of bias | Inconsistency | Indirectness | Imprecision | Other considerations | Intervention | Comparison | Relative (95% CI) | Absolute (95% CI) | | |
| **ADL（Barthel index）** | | | | | | | | | | | | |
| 2 | randomised trials | very serious[a] | serious[b] | not serious | very serious[c] | none | 54 | 52 | - | MD **10.76 higher** (12.95 lower to 34.48 higher) | ⊕◯◯◯ Very low | CRITICAL |
| **Exercise tolerance (6MWD)** | | | | | | | | | | | | |
| **0** | | | | | | | | | | | | CRITICAL |
| **Muscle strength (MRC SS)** | | | | | | | | | | | | |
| 2 | randomised trials | serious[d] | serious[e] | not serious | very serious[c] | none | 30 | 38 | - | MD **4.68 higher** (2.66 lower to 12.03 higher) | ⊕◯◯◯ Very low | CRITICAL |
| **Muscle mass** | | | | | | | | | | | | |
| 2 | randomised trials | not serious | serious[f] | not serious | very serious[c] | none | 19 | 23 | - | MD **0.37 mm higher** (2.57 lower to 3.30 higher) | ⊕◯◯◯ Very low | CRITICAL |
| **Ventilator days** | | | | | | | | | | | | |
| 10 | randomised trials | not serious | not serious | not serious | serious[g] | none | 249 | 253 | - | MD **1 day lower** (2.18 lower to 0.18 higher) | ⊕⊕⊕◯ Moderate | CRITICAL |
| **Hospital days** | | | | | | | | | | | | |
| 7 | randomised trials | serious[h] | serious[i] | not serious | serious[g] | none | 197 | 214 | - | MD **3.77 day lower** (7.98 lower to 0.43 higher) | ⊕◯◯◯ Very low | CRITICAL |
| **Adverse events** | | | | | | | | | | | | |
| 4 | randomised trials | serious[j] | very serious[k] | not serious | very serious[l] | none | 11/65 (16.9%) | 18/74 (24.3%) | not estimable | **140 fewer per 1,000** (from 380 fewer to 100 more) | ⊕◯◯◯ Very low | CRITICAL |

**CI:** confidence interval; **MD:** mean difference

## Explanations

a. Downgrade two level due to risk of bias (blinding of participants and personnel, blinding outcome assessment, incomplete outcome data, and selective reporting).
b. Downgrade one level due to substantial heterogeneity (I2=64%).
c. Downgrade two level due to the 95% confidence interval overlaps 0 and smaller than OIS.
d. Downgrade one level due to risk of bias (blinding of participants and personnel, blinding outcome assessment, and incomplete outcome data).
e. Downgrade one level due to substantial heterogeneity (I2=73%).
f. Downgrade one level due to substantial heterogeneity (I2=47%).
g. Downgrade one level due to the 95% confidence interval overlaps 0.
h. Downgrade one level due to risk of bias (blinding of participants and personnel, and incomplete outcome data).
i. Downgrade one level due to substantial heterogeneity (I2=52%).
j. Downgrade one level due to risk of bias (blinding of participants and personnel, blinding outcome assessment, incomplete outcome data, and selective reporting).
k. Downgrade two level due to considerable heterogeneity (I2=87%).
l. Downgrade two level due to the 95% confidence interval overlaps 1 and smaller than OIS.

| | JUDGEMENT | | | | | | |
|---|---|---|---|---|---|---|---|
| **PROBLEM** | No | Probably no | **Probably yes** | Yes | | Varies | Don't know |
| **DESIRABLE EFFECTS** | Trivial | Small | **Moderate** | Large | | Varies | Don't know |
| **UNDESIRABLE EFFECTS** | Large | Moderate | Small | **Trivial** | | Varies | Don't know |
| **CERTAINTY OF EVIDENCE** | Very low | Low | **Moderate** | High | | | No included studies |
| **VALUES** | Important uncertainty or variability | Possibly important uncertainty or variability | **Probably no important uncertainty or variability** | No important uncertainty or variability | | | |
| **BALANCE OF EFFECTS** | Favors the comparison | Probably favors the comparison | Does not favor either the intervention or the comparison | Probably favors the intervention | **Favors the intervention** | Varies | Don't know |
| **ACCEPTABILITY** | No | Probably no | Probably yes | Yes | | **Varies** | Don't know |
| **FEASIBILITY** | No | Probably no | Probably yes | Yes | | **Varies** | Don't know |

CQ3-2
P: Patient with critical illness
I: In-bed cycle ergometry
C: Non-in-bed cycle ergometry

| Certainty assessment | | | | | | | № of patients | | Effect | | Certainty | Importance |
|---|---|---|---|---|---|---|---|---|---|---|---|---|
| № of studies | Study design | Risk of bias | Inconsistency | Indirectness | Imprecision | Other considerations | Intervention | Comparison | Relative (95% CI) | Absolute (95% CI) | | |
| **ADL (Barthel index)** | | | | | | | | | | | | |
| **0** | | | | | | | | | | | | CRITICAL |
| **Exercise tolerance (6MWD)** | | | | | | | | | | | | |
| 1 | randomised trials | very serious[a] | not serious | not serious | very serious[b] | none | 31 | 36 | - | MD **53 m higher** (16.85 lower to 122.85 higher) | ⊕◯◯◯ Very low | CRITICAL |
| **Muscle strength (MRC SS)** | | | | | | | | | | | | |
| 2 | randomised trials | serious[c] | not serious | not serious | serious[d] | none | 58 | 52 | - | MD **0.19 lower** (2.91 lower to 2.53 higher) | ⊕⊕◯◯ Low | CRITICAL |
| **Muscle mass** | | | | | | | | | | | | |
| 1 | randomised trials | not serious | not serious | not serious | very serious[e] | none | 12 | 12 | - | MD **2.75 mm higher** (4.17 lower to 9.67 higher) | ⊕⊕◯◯ Low | CRITICAL |
| **Ventilator days** | | | | | | | | | | | | |
| 7 | randomised trials | serious[f] | not serious | not serious | serious[g] | none | 166 | 153 | - | MD **0.76 day higher** (0.69 lower to 2.2 higher) | ⊕⊕◯◯ Low | CRITICAL |
| **Hospital days** | | | | | | | | | | | | |
| 6 | randomised trials | serious[h] | not serious | not serious | serious[i] | none | 146 | 131 | - | MD **1.28 day lower** (5.44 lower to 2.88 higher) | ⊕⊕◯◯ Low | CRITICAL |
| **Adverse events** | | | | | | | | | | | | |
| 1 | randomised trials | very serious[j] | not serious | not serious | very serious[k] | none | 0/31 (0.0%) | 0/36 (0.0%) | not estimable | **0 fewer per 1,000** (from 60 fewer to 60 more) | ⊕◯◯◯ Very low | CRITICAL |

**CI:** confidence interval; **MD:** mean difference

## Explanations

a. Downgrade two level due to risk of bias (blinding of participants and personnel, blinding of outcome assessment, Incomplete outcome data, and other bias).
b. Downgrade two levels due to wide range of 95% confidence interval and smaller than OIS.
c. Downgrade one level due to risk of bias (blinding of participants and personnel).
d. Downgrade one level due to smaller than OIS.
e. Downgrade two level due to smaller than OIS and the 95% CI overlaps 0.
f. Downgraded one level due to risk of bias (blinding of participants, personnel, Incomplete outcome data, and selective reporting).
g. Downgrade one level due to smaller than OIS.
h. Downgrade one level due to risk of bias (blinding of participants and personnel, Incomplete outcome data).
i. Downgrade one level due to smaller than OIS.
j. Downgrade two level due to risk of bias (blinding of participants and personnel, blinding of outcome assessment, Incomplete outcome data, and other bias).
k. Downgraded two level due to the 95% confidence interval overlaps 1 and small number of studies.

| | JUDGEMENT | | | | | | |
|---|---|---|---|---|---|---|---|
| **PROBLEM** | No | Probably no | **Probably yes** | Yes | | Varies | Don't know |
| **DESIRABLE EFFECTS** | Trivial | **Small** | Moderate | Large | | Varies | Don't know |
| **UNDESIRABLE EFFECTS** | Large | Moderate | Small | **Trivial** | | Varies | Don't know |
| **CERTAINTY OF EVIDENCE** | **Very low** | Low | Moderate | High | | | No included studies |
| **VALUES** | Important uncertainty or variability | Possibly important uncertainty or variability | **Probably no important uncertainty or variability** | No important uncertainty or variability | | | |
| **BALANCE OF EFFECTS** | Favors the comparison | Probably favors the comparison | Does not favor either the intervention or the comparison | **Probably favors the intervention** | Favors the intervention | Varies | Don't know |
| **ACCEPTABILITY** | No | Probably no | Probably yes | Yes | | **Varies** | Don't know |
| **FEASIBILITY** | No | Probably no | Probably yes | Yes | | **Varies** | Don't know |

CQ 3-3
P: Patient with critical illness
I: In-bed cycle ergometry and neuromuscular electrical stimulation
C: Non-in-bed cycle ergometry and neuromuscular electrical stimulation

| Certainty assessment | | | | | | | № of patients | | Effect | | Certainty | Importance |
|---|---|---|---|---|---|---|---|---|---|---|---|---|
| № of studies | Study design | Risk of bias | Inconsistency | Indirectness | Imprecision | Other considerations | Intervention | Comparison | Relative (95% CI) | Absolute (95% CI) | | |
| **ADL (Katz Index, Barthel index)** | | | | | | | | | | | | |
| 2 | randomised trials | serious[a] | serious[b] | not serious | very serious[c] | none | 118 | 132 | - | SMD **0.21 higher** (0.29 lower to 0.71 higher) | ⊕◯◯◯ Very low | CRITICAL |
| **Exercise tolerance (6MWD)** | | | | | | | | | | | | |
| 1 | randomised trials | not serious | not serious | not serious | serious[d] | none | 23 | 23 | - | MD **81 m higher** (7.01 higher to 154.99 higher) | ⊕⊕⊕◯ Moderate | CRITICAL |
| **Muscle strength (MRC SS)** | | | | | | | | | | | | |
| 3 | randomised trials | not serious | very serious[e] | not serious | serious[f] | none | 235 | 242 | - | MD **0.47 higher** (4.09 lower to 5.04 higher) | ⊕◯◯◯ Very low | CRITICAL |
| **Muscle mass** | | | | | | | | | | | | |
| 3 | randomised trials | serious[g] | serious[h] | not serious | serious[i] | none | 295 | 290 | - | SMD **0.39 higher** (0.13 higher to 0.65 higher) | ⊕◯◯◯ Very low | CRITICAL |
| **Ventilator days** | | | | | | | | | | | | |
| 2 | randomised trials | serious[j] | not serious | not serious | serious[k] | none | 238 | 236 | - | MD **0 day** (0.25 lower to 0.25 higher) | ⊕⊕◯◯ Low | CRITICAL |
| **Hospital days** | | | | | | | | | | | | |
| 2 | randomised trials | not serious | not serious | not serious | serious[l] | none | 150 | 151 | - | MD **1.96 day lower** (3.32 lower to 0.6 lower) | ⊕⊕⊕◯ Moderate | CRITICAL |
| **Adverse events** | | | | | | | | | | | | |
| 1 | randomised trials | very serious[m] | not serious | not serious | very serious[n] | none | 7/158 (4.4%) | 9/154 (5.8%) | not estimable | **10 fewer per 1,000** (from 60 fewer to 30 more) | ⊕◯◯◯ Very low | CRITICAL |

**CI:** confidence interval; **MD:** mean difference; **RR:** risk ratio; **SMD:** standardised mean difference

## Explanations

a. Downgrade one level due to risk of bias (blinding of participants and personnel).
b. Downgrade one level due to considerable heterogeneity (I2 = 65%).
c. Downgrade two level due to wide range of 95% confidence interval and smaller than OIS.
d. Downgrade one level due to smaller than OIS.
e. Downgrade two level due to considerable heterogeneity (I2 = 98%).
f. Downgrade one level due to smaller than OIS.
g. Downgrade one level due to risk of bias (blinding of participants and personnel).
h. Downgrade one level due to considerable heterogeneity (I2 = 58%).
i. Downgrade one level due to smaller than OIS.
j. Downgrade one level due to risk of bias (blinding of participants and personnel).
k. Downgrade one level due to smaller than OIS.
l. Downgrade one level due to smaller than OIS.
m. Downgrade two level due to risk of bias (blinding of participants and personnel, blinding of outcome assessment, Incomplete outcome data, and Selective reporting).
n. Downgrade two levels due to wide range of 95% confidence interval and smaller than OIS.

| | JUDGEMENT | | | | | | |
|---|---|---|---|---|---|---|---|
| PROBLEM | No | Probably no | **Probably yes** | Yes | | Varies | Don't know |
| DESIRABLE EFFECTS | Trivial | Small | **Moderate** | Large | | Varies | Don't know |
| UNDESIRABLE EFFECTS | Large | Moderate | Small | **Trivial** | | Varies | Don't know |
| CERTAINTY OF EVIDENCE | Very low | Low | **Moderate** | High | | | No included studies |
| VALUES | Important uncertainty or variability | Possibly important uncertainty or variability | **Probably no important uncertainty or variability** | No important uncertainty or variability | | | |
| BALANCE OF EFFECTS | Favors the comparison | Probably favors the comparison | Does not favor either the intervention or the comparison | Probably favors the intervention | **Favors the intervention** | Varies | Don't know |
| ACCEPTABILITY | No | Probably no | Probably yes | Yes | | **Varies** | Don't know |
| FEASIBILITY | No | Probably no | Probably yes | Yes | | **Varies** | Don't know |

CQ 5
P: Patient with critical illness
I: Dysphagia management based on video endoscopic examination
C: Dysphagia management based on usual assessment

| Quality assessment | | | | | | | № of patients | | Effect | | Quality | Importance |
|---|---|---|---|---|---|---|---|---|---|---|---|---|
| № of studies | Study design | Risk of bias | Inconsistency | Indirectness | Imprecision | Other considerations | Intervention | Comparison | Relative (95% CI) | Absolute (95% CI) | | |
| **Pneumonia** | | | | | | | | | | | | |
| 1 | randomised trials | serious [a] | not serious | not serious | very serious [b] | none | 5/37 (13.5%) | 2/33 (6.1%) | **RR 2.23** (0.46 to 10.73) | **75 more per 1,000** (from 33 fewer to 590 more) | ⨁◯◯◯ Very low | CRITICAL |
| **Adverse events** | | | | | | | | | | | | |
| 1 | randomised trials | serious [a] | not serious | not serious | very serious [b] | none | 2/37 (5.4%) | 1/33 (3.0%) | **RR 1.78** (0.17 to 18.78) | **24 more per 1,000** (from 25 fewer to 539 more) | ⨁◯◯◯ Very low | CRITICAL |

a. Downgraded because a randomized trial did not blind patients, caretakers and investigators.
b. Downgraded because the number of events was very small and *the 95% confidence interval* includes both clinically thresholds for benefits and harms.

| | JUDGEMENT | | | | | | |
|---|---|---|---|---|---|---|---|
| PROBLEM | No | Probably no | Probably yes | **Yes** | | Varies | Don't know |
| DESIRABLE EFFECTS | Trivial | Small | Moderate | Large | | Varies | **Don't know** |
| UNDESIRABLE EFFECTS | Large | Moderate | **Small** | Trivial | | Varies | Don't know |
| CERTAINTY OF EVIDENCE | **Very low** | Low | Moderate | High | | | No included studies |
| VALUES | Important uncertainty or variability | Possibly important uncertainty or variability | Probably no important uncertainty or variability | **No important uncertainty or variability** | | | |
| BALANCE OF EFFECTS | Favors the comparison | **Probably favors the comparison** | Does not favor either the intervention or the comparison | Probably favors the intervention | Favors the intervention | Varies | Don't know |
| ACCEPTABILITY | No | Probably no | **Probably yes** | Yes | | Varies | Don't know |
| FEASIBILITY | No | Probably no | **Probably yes** | Yes | | Varies | Don't know |

CQ 6
P: Patient with critical illness
I: Dysphagia rehabilitation
C: No intervention

| Certainty assessment | | | | | | | № of patients | | Effect | | Certainty | Importance |
|---|---|---|---|---|---|---|---|---|---|---|---|---|
| № of studies | Study design | Risk of bias | Inconsistency | Indirectness | Imprecision | Other considerations | Intervention | Comparison | Relative (95% CI) | Absolute (95% CI) | | |
| **Mortality** | | | | | | | | | | | | |
| 9 | randomised trials | not serious | not serious | very serious[a] | very serious[b] | none | 47/353 (13.3%) | 29/238 (12.2%) | **RR 0.99** (0.55 to 1.78) | **1 fewer per 1,000** (from 55 fewer to 95 more) | ⨁◯◯◯ Very low | CRITICAL |
| **Pneumonia** | | | | | | | | | | | | |
| 5 | randomised trials | not serious | not serious | serious[c] | serious[d] | none | 71/299 (23.7%) | 73/201 (36.3%) | **RR 0.60** (0.46 to 0.78) | **145 fewer per 1,000** (from 196 fewer to 80 fewer) | ⨁⨁◯◯ Low | CRITICAL |
| **QOL (SF-36, EQ-5D)** | | | | | | | | | | | | |
| 0 | randomised trials | | | | | | | | | **-** | | CRITICAL |
| **ADL (FIM, Barthel Index, Katz Index)** | | | | | | | | | | | | |
| 0 | | | | | | | | | | **-** | | CRITICAL |
| **Eating Status (FOIS)** | | | | | | | | | | | | |
| 3 | randomised trials | not serious | serious[e] | serious[c] | very serious[f] | none | 71 | 70 | - | MD **0.79 higher** (0.21 lower to 1.79 higher) | ⨁◯◯◯ Very low | CRITICAL |
| **Length of Hospital Stay** | | | | | | | | | | | | |
| 3 | randomised trials | not serious | serious[g] | serious[c] | very serious[f] | none | 253 | 142 | - | MD **0.26 day higher** (3.95 lower to 4.47 higher) | ⨁◯◯◯ Very low | IMPORTANT |
| **Adverse events** | | | | | | | | | | | | |
| 4 | randomised trials | not serious | serious[h] | serious[i] | very serious[j] | none | 100/266 (37.6%) | 67/153 (43.8%) | **RR 0.97** (0.40 to 2.31) | **13 fewer per 1,000** (from 263 fewer to 574 more) | ⨁◯◯◯ Very low | CRITICAL |

**CI:** confidence interval; **MD:** mean difference; **RR:** risk ratio

## Explanations

a. Downgraded by two points because the definition and timing of mortality in the included studies potentially varied and most of the studies focused on stroke patients who might have partly differed from critically ill ones.
b. Downgraded by two points because the optimal information size was not reached and the confidence interval of the effect size straddled 1.
c. Downgraded by one point because most of the studies focused on stroke patients, who might have partly differed from critically ill ones.
d. Downgraded by one point because the optimal information size was not reached.
e. Downgraded by one point due to moderate heterogeneity (I2= 53%).
f. Downgraded by two points because the optimal information size was not reached and the 95% confidence interval of the pooled effect straddled 0.
g. Downgraded by one point due to the substantial heterogeneity (I2= 63%).
h. Downgraded by one point due to moderate heterogeneity (I2= 54%).
i. Downgraded by one point because most of the studies focused on stroke patients, who might have partly differed from critically ill patients.

j. Downgraded by two points because the optimal information size was not reached and the confidence interval of the effect size straddled 1.

| | JUDGEMENT | | | | | | |
|---|---|---|---|---|---|---|---|
| **PROBLEM** | No | Probably no | Probably yes | Yes | | Varies | Don't know |
| **DESIRABLE EFFECTS** | Trivial | Small | Moderate | Large | | Varies | Don't know |
| **UNDESIRABLE EFFECTS** | Large | Moderate | Small | Trivial | | Varies | Don't know |
| **CERTAINTY OF EVIDENCE** | Very low | Low | Moderate | High | | | No included studies |
| **VALUES** | Important uncertainty or variability | Possibly important uncertainty or variability | Probably no important uncertainty or variability | No important uncertainty or variability | | | |
| **BALANCE OF EFFECTS** | Favors the comparison | Probably favors the comparison | Does not favor either the intervention or the comparison | Probably favors the intervention | Favors the intervention | Varies | Don't know |
| **ACCEPTABILITY** | No | Probably no | Probably yes | Yes | | Varies | Don't know |
| **FEASIBILITY** | No | Probably no | Probably yes | Yes | | Varies | Don't know |

CQ 9
P: Adult patients with critical illness
I: Energy provision of equal to or more than 20 kcal/kg/day or 70 % energy expenditure
C: Energy provision of less than 20 kcal/kg/day or 70 % energy expenditure, or usual nutrition therapy
Energy amount was determined as target energy provision in the study design. Target energy was identified between 4 and 10 days from admission. Actual body weight or adjusted body weight were used.

| Certainty assessment | | | | | | | № of patients | | Effect | | Certainty | Importance |
|---|---|---|---|---|---|---|---|---|---|---|---|---|
| № of studies | Study design | Risk of bias | Inconsistency | Indirectness | Imprecision | Other considerations | Intervention | Comparison | Relative (95% CI) | Absolute (95% CI) | | |
| **ADL score** | | | | | | | | | | | | |
| 0 | | | | | | | | | | - | | CRITICAL |
| **Hand grip strength** | | | | | | | | | | | | |
| 2 | randomised trials | serious[a] | not serious | not serious | very serious[b] | none | 93 | 99 | - | MD **0.58 kg higher** (4.77 lower to 5.92 higher) | ⨁◯◯◯ Very low | CRITICAL |
| **Muscle volume change** | | | | | | | | | | | | |
| 0 | | | | | | | | | | | | CRITICAL |
| **QOL: ED-5D-3L** | | | | | | | | | | | | |
| 2 | randomised trials | very serious[c] | not serious | not serious | very serious[b] | none | 263 | 288 | - | MD **0.01 higher** (0.03 lower to 0.05 higher) | ⨁◯◯◯ Very low | CRITICAL |
| **Diarrhea** | | | | | | | | | | | | |
| 3 | randomised trials | not serious | Serious[d] | not serious | very serious[e] | none | 121/560 (21.6%) | 101/554 (18.2%) | **RR 1.20** (0.95 to 1.51) | **36 more per 1,000** (from 9 fewer to 93 more) | ⨁◯◯◯ Very low | CRITICAL |
| **Mortality** | | | | | | | | | | | | |
| 8 | randomised trials | not serious | not serious | not serious | serious[f] | none | 341/1369 (24.9%) | 339/1385 (24.6%) | **RR 1.01** (0.89 to 1.15) | **0 fewer per 1,000** (from 30 fewer to 34 more) | ⨁⨁⨁◯ Moderate | IMPORTANT |
| **Hospital day** | | | | | | | | | | | | |
| 7 | randomised trials | not serious | serious[g] | not serious | very serious[e] | none | 401 | 391 | - | MD **1.08 day lower** (4.86 lower to 2.7 higher) | ⨁◯◯◯ Very low | IMPORTANT |

**CI:** confidence interval; **MD:** mean difference; **RR:** risk ratio; **SMD:** standardised mean difference

## Explanations

a. Downgraded one level due to high risk of bias (difficulty in blinding patients and providers, incomplete outcomes)
b. Many have small sample sizes where the 95% CI includes no effect and does not meet OIS
c. Downgraded 2 levels due to subjective outcome and very high risk of bias (difficulty in blinding patients and providers, unknown blinding of assessors, incomplete outcome)

d. I2=55%, one step downgrade
e. 95% CI not including no effect, less than OIS
f. Meets OIS but 95% CI includes no effect
g. I2=45%, one step downgrade

| | JUDGEMENT | | | | | | |
|---|---|---|---|---|---|---|---|
| **PROBLEM** | No | Probably no | **Probably yes** | Yes | | Varies | Don't know |
| **DESIRABLE EFFECTS** | **Trivial** | Small | Moderate | Large | | Varies | Don't know |
| **UNDESIRABLE EFFECTS** | Large | Moderate | Small | **Trivial** | | Varies | Don't know |
| **CERTAINTY OF EVIDENCE** | **Very low** | Low | Moderate | High | | | No included studies |
| **VALUES** | Important uncertainty or variability | Possibly important uncertainty or variability | **Probably no important uncertainty or variability** | No important uncertainty or variability | | | |
| **BALANCE OF EFFECTS** | Favors the comparison | Probably favors the comparison | **Does not favor either the intervention or the comparison** | Probably favors the intervention | Favors the intervention | Varies | Don't know |
| **ACCEPTABILITY** | No | Probably no | Probably yes | **Yes** | | Varies | Don't know |
| **FEASIBILITY** | No | Probably no | Probably yes | **Yes** | | Varies | Don't know |

CQ 10
P: Adult patients with critical illness
I: Protein provision of equal to or more than 1 g/kg/day
C: Protein provision of less than 1 g/kg/day 1g/kg/day, or usual nutrition therapy
Protein amount was determined as target protein provision in the study design. Target protein was identified between 4 and 10 days from admission. Actual body weight or adjusted body weight were used.

| Certainty assessment | | | | | | | № of patients | | Effect | | Certainty | Importance |
|---|---|---|---|---|---|---|---|---|---|---|---|---|
| № of studies | Study design | Risk of bias | Inconsistency | Indirectness | Imprecision | Other considerations | Intervention | Comparison | Relative (95% CI) | Absolute (95% CI) | | |
| **Barthel index** | | | | | | | | | | | | |
| 3 | randomised trials | not serious | serious[a] | not serious | very serious[b] | none | 114 | 122 | - | MD **21.55 higher** (1.3 lower to 44.4 higher) | ⊕◯◯◯ Very low | CRITICAL |
| **hand grip strength** | | | | | | | | | | | | |
| 2 | randomised trials | very serious[c] | not serious | not serious | very serious[d] | none | 25 | 40 | - | MD **1 kg lower** (5.79 lower to 3.79 higher) | ⊕◯◯◯ Very low | CRITICAL |
| **muscle volume change** | | | | | | | | | | | | |
| 3 | randomised trials | not serious | not serious | not serious | serious[e] | none | 145 | 141 | - | SMD **0.47 higher** (0.24 higher to 0.71 higher) | ⊕⊕⊕◯ Moderate | CRITICAL |
| **QOL score (physical )** | | | | | | | | | | | | |
| 3 | randomised trials | very serious[f] | not serious | not serious | serious[g] | none | 361 | 352 | - | SMD **0.13 lower** (0.31 lower to 0.06 higher) | ⊕◯◯◯ Very low | CRITICAL |
| **Diarrhea** | | | | | | | | | | | | |
| 7 | randomised trials | not serious | serious[h] | not serious | very serious[i] | none | 93/234 (39.7%) | 104/231 (45.0%) | **RR 0.90** (0.61 to 1.31) | **45 fewer per 1,000** (from 176 fewer to 140 more) | ⊕◯◯◯ Very low | CRITICAL |
| **Mortality** | | | | | | | | | | | | |
| 11 | randomised trials | not serious | not serious | not serious | very serious[j] | none | 128/750 (17.1%) | 149/778 (19.2%) | **RR 0.90** (0.73 to 1.12) | **19 fewer per 1,000** (from 52 fewer to 23 more) | ⊕⊕◯◯ Low | IMPORTANT |
| **hospital day** | | | | | | | | | | | | |
| 13 | randomised trials | not serious | serious[k] | not serious | not serious | none | 812 | 832 | - | MD **0.36 day higher** (0.98 lower to 1.7 higher) | ⊕⊕⊕◯ Moderate | IMPORTANT |

**CI:** confidence interval; **MD:** mean difference; **RR:** risk ratio; **SMD:** standardized mean difference

## Explanations

a. I2 is high at 76%. However, the forest plot was the same direction. Therefore, downgrage was limitted to serious.
b. Downgraded due to total sample size of 236<800
c. Included two studies had high risk of bias about blinding of participants and personeel and incomplete outcome data.
d. Downgraded due to total sample size of 65<800
e. Downgraded due to total sample size of 286<800
f. No study was judged to be low risk of bias about blinding of participants and personeel and blinding of outcome assessment.
g. Downgraded due to total sample size of 712<800
h. I2 is high at 64%. Forest plot direction a little bit different among included studies.
i. Downgraded due to total events size of 197<300
j. Downgraded due to total events size of 277<300
k. I2 is high at 61%.

| | JUDGEMENT | | | | | | |
|---|---|---|---|---|---|---|---|
| **PROBLEM** | No | Probably no | **Probably yes** | Yes | | Varies | Don't know |
| **DESIRABLE EFFECTS** | Trivial | **Small** | Moderate | Large | | Varies | Don't know |
| **UNDESIRABLE EFFECTS** | Large | Moderate | Small | **Trivial** | | Varies | Don't know |
| **CERTAINTY OF EVIDENCE** | **Very low** | Low | Moderate | High | | | No included studies |
| **VALUES** | Important uncertainty or variability | Possibly important uncertainty or variability | **Probably no important uncertainty or variability** | No important uncertainty or variability | | | |
| **BALANCE OF EFFECTS** | Favors the comparison | Probably favors the comparison | Does not favor either the intervention or the comparison | **Probably favors the intervention** | Favors the intervention | Varies | Don't know |
| **ACCEPTABILITY** | No | Probably no | Probably yes | **Yes** | | Varies | Don't know |
| **FEASIBILITY** | No | Probably no | Probably yes | **Yes** | | Varies | Don't know |

CQ 11
P: Critically ill children admitted to the ICU
I: Implementation of early physical rehabilitation protocol
C: No intervention

| Certainty assessment | | | | | | | № of patients | | Effect | | Certainty | Importance |
|---|---|---|---|---|---|---|---|---|---|---|---|---|
| № of studies | Study design | Risk of bias | Inconsistency | Indirectness | Imprecision | Other considerations | Intervention | Comparison | Relative (95% CI) | Absolute (95% CI) | | |
| **Mortality** | | | | | | | | | | | | |
| 1 | randomised trials | | | | | | | | - | - | - | CRITICAL |
| **Length of Hospital Stay** | | | | | | | | | | | | |
| 1 | randomised trials | not serious | not serious | not serious | very serious[a] | none | 26 | 32 | - | MD **0 day** (4.98 lower to 4.98 higher) | ⨁⨁◯◯ Low | CRITICAL |
| **Length of ICU Stay** | | | | | | | | | | | | |
| 1 | randomised trials | not serious | not serious | not serious | very serious[a] | none | 26 | 32 | - | MD **1.5 day higher** (2.63 lower to 5.63 higher) | ⨁⨁◯◯ Low | CRITICAL |
| **Adverse events requiring therapeutic intervention** | | | | | | | | | | | | |
| 2 | randomised trials | not serious | not serious | not serious | Very serious[a] | none | 1/46 (2.2%) | 4/42 (9.5%) | **RR 0.31** (0.04 to 2.59) | **66 fewer per 1,000** (from 91 fewer to 151 more) | ⨁⨁◯◯ Low | CRITICAL |

**CI:** confidence interval; **RR:** risk ratio

## Explanations

a: Downgraded two level due to imprecision (wide confidence intervals and the small sample size).

| | JUDGEMENT | | | | | | |
|---|---|---|---|---|---|---|---|
| PROBLEM | No | Probably no | **Probably yes** | Yes | | Varies | Don't know |
| DESIRABLE EFFECTS | Trivial | Small | Moderate | Large | | Varies | **Don't know** |
| UNDESIRABLE EFFECTS | Large | Moderate | Small | **Trivial** | | Varies | Don't know |
| CERTAINTY OF EVIDENCE | **Very low** | Low | Moderate | High | | | No included studies |
| VALUES | Important uncertainty or variability | Possibly important uncertainty or variability | Probably no important uncertainty or variability | **No important uncertainty or variability** | | | |
| BALANCE OF EFFECTS | Favors the comparison | Probably favors the comparison | Does not favor either the intervention or the comparison | Probably favors the intervention | Favors the intervention | Varies | **Don't know** |
| ACCEPTABILITY | No | Probably no | **Probably yes** | Yes | | Varies | Don't know |
| FEASIBILITY | No | Probably no | **Probably yes** | Yes | | Varies | Don't know |

CQ12
P: Critically ill children who received mechanical ventilation including NPPV
I: Respiratory physical therapy
C: No intervention or standard care

| Certainty assessment | | | | | | | № of patients | | Effect | | Certainty | Importance |
|---|---|---|---|---|---|---|---|---|---|---|---|---|
| № of studies | Study design | Risk of bias | Inconsistency | Indirectness | Imprecision | Other considerations | Intervention | Comparison | Relative (95% CI) | Absolute (95% CI) | | |
| **Mortality** | | | | | | | | | | | | |
| 2[a] | randomised trials | serious[b] | not serious | not serious | serious[c] | none | 9/73 (12.3%) | 16/70 (22.9%) | **RR 0.53** (0.22 to 1.28) | **107 fewer per 1,000** (from 178 fewer to 64 more) | ⊕⊕◯◯ Low | CRITICAL |
| **Length of Hospital Stay** | | | | | | | | | | | | |
| 0 | | | | | | | | | | | | CRITICAL |
| **Length of ICU Stay** | | | | | | | | | | | | |
| 0 | | | | | | | | | | | | CRITICAL |
| **Duration of mechanical ventilation** | | | | | | | | | | | | |
| 1[a] | randomised trials | serious[b] | not serious | not serious | very serious[d] | none | 22 | 20 | - | MD **10.1 hours higher** (37.57 lower to 57.77 higher) | ⊕◯◯◯ Very low | CRITICAL |
| **Adverse events requiring treatment** | | | | | | | | | | | | |
| 1[a] | randomised trials | very serious[e] | not serious | not serious | serious[c] | none | 4/51 (7.8%) | 5/50 (10.0%) | **RR 0.78** (0.22 to 2.75) | **22 fewer per 1,000** (from 78 fewer to 175 more) | ⊕◯◯◯ Very low | CRITICAL |
| **Oxygenation（PaO2/FIO2 ratio）** | | | | | | | | | | | | |
| 2[a] | randomised trials | serious[b] | serious[f] | not serious | serious[c] | none | 72 | 69 | - | MD **67.87 higher** (96.35 lower to 232.09 higher) | ⊕◯◯◯ Very low | IMPORTANT |
| **Atelectasis** | | | | | | | | | | | | |
| 0 | | | | | | | | | | | | IMPORTANT |

**CI:** confidence interval; **MD:** mean difference; **RR:** risk ratio

## Explanations

a. All the included studies investigated prone positioning as physical therapy
b. Downgraded one level due to the risk of bias (allocation concealment, selective reporting and other bias).
c. Downgraded one level due to smaller than OIS.

d. Downgrade two levels due to wide range of 95% confidence interval and smaller than OIS.
e. Downgraded two levels due to insufficient information about the number of the patient who experienced adverse events and following selection of unplanned extubation as a representative adverse event by reviewers.
f. Downgraded one level because substantial heterogeneity (I2 = 68%)

| | JUDGEMENT | | | | | | |
|---|---|---|---|---|---|---|---|
| **PROBLEM** | No | Probably no | Probably yes | **Yes** | | Varies | Don't know |
| **DESIRABLE EFFECTS** | Trivial | Small | Moderate | Large | | Varies | Don't know |
| **UNDESIRABLE EFFECTS** | Large | Moderate | Small | Trivial | | Varies | Don't know |
| **CERTAINTY OF EVIDENCE** | Very low | Low | Moderate | High | | | No included studies |
| **VALUES** | Important uncertainty or variability | Possibly important uncertainty or variability | Probably no important uncertainty or variability | No important uncertainty or variability | | | |
| **BALANCE OF EFFECTS** | Favors the comparison | Probably favors the comparison | Does not favor either the intervention or the comparison | Probably favors the intervention | Favors the intervention | Varies | Don't know |
| **ACCEPTABILITY** | No | Probably no | Probably yes | Yes | | Varies | Don't know |
| **FEASIBILITY** | No | Probably no | Probably yes | Yes | | Varies | Don't know |

CQ13
P: Patient with critical illness
I: Enhanced rehabilitation following ICU discharge
C: No intervention or usual care

| Certainty assessment | | | | | | | № of patients | | Effect | | Certainty | Importance |
|---|---|---|---|---|---|---|---|---|---|---|---|---|
| № of studies | Study design | Risk of bias | Inconsistency | Indirectness | Imprecision | Other considerations | Intervention | Comparison | Relative (95% CI) | Absolute (95% CI) | | |
| **Quality of life (physical)** | | | | | | | | | | | | |
| 9 | randomised trials | very serious[a,b] | not serious | not serious | serious[c] | none | 398 | 409 | - | SMD **0.1 higher** (0.06 lower to 0.25 higher) | ⊕◯◯◯ Very low | CRITICAL |
| **Quality of life (mental)** | | | | | | | | | | | | |
| 9 | randomised trials | very serious[a,b] | serious[d] | not serious | serious[c] | publication bias strongly suspected[e] | 395 | 408 | - | SMD **0.19 higher** (0.03 lower to 0.42 higher) | ⊕◯◯◯ Very low | CRITICAL |
| **Quality of life（overall）** | | | | | | | | | | | | |
| 5 | randomised trials | very serious[a,b] | serious[f] | not serious | very serious[c,g] | none | 201 | 223 | - | SMD **0.22 SD higher** (0.09 lower to 0.54 higher) | ⊕◯◯◯ Very low | CRITICAL |
| **Mortality** | | | | | | | | | | | | |
| 2 | randomised trials | serious[b] | not serious | not serious | very serious[g,h] | none | 10/148 (6.8%) | 7/140 (5.0%) | RR 1.44 (0.39 to 5.36) | **22 more per 1,000** (from 31 fewer to 218 more) | ⊕◯◯◯ Very low | CRITICAL |
| **Activities of daily living** | | | | | | | | | | | | |
| 2 | randomised trials | serious[b] | serious[i] | not serious | very serious[c,f] | none | 53 | 62 | - | SMD **0.41 SD lower** (1.28 lower to 0.46 higher) | ⊕◯◯◯ Very low | CRITICAL |
| **Return to work** | | | | | | | | | | | | |
| 0 | | | | | | | | | | | | CRITICAL |
| **All adverse events** | | | | | | | | | | | | |
| 4 | randomised trials | very serious[a,j] | not serious | not serious | very serious[c,h] | none | 6/84 (7.1%) | 2/82 (2.4%) | RR 3.00 (0.66 to 13.69) | **49 more per 1,000** (from 8 fewer to 310 more) | ⊕◯◯◯ Very low | CRITICAL |

CI: confidence interval; RR: risk ratio; SMD: standardised mean difference

Explanations

a. Downgraded one level due to risk of bias (blinding of outcome assessment)
b. Downgraded one level due to risk of bias (blinding of participants and personnel).
c. Downgraded one level due to a smaller sample size than OIS.
d. Downgraded one level due to substantial heterogeneity (I2 = 53%).
e. Downgraded one level because the asymmetry of the funnel plot is suspected.
f. Downgraded one level due to substantial heterogeneity (I2 = 52%).
g. Downgraded one level due to a wide range of 95% confidence interval and straddling zero.
h. Downgraded one level due to wide range of 95% confidence interval.
i. Downgraded one level due to considerable heterogeneity (I2 = 81%).
j. Downgraded one level due to risk of bias (Selective reporting).

| | JUDGEMENT | | | | | | |
|---|---|---|---|---|---|---|---|
| PROBLEM | No | Probably no | Probably yes | Yes | | Varies | Don't know |
| DESIRABLE EFFECTS | Trivial | Small | Moderate | Large | | Varies | Don't know |
| UNDESIRABLE EFFECTS | Large | Moderate | Small | Trivial | | Varies | Don't know |
| CERTAINTY OF EVIDENCE | Very low | Low | Moderate | High | | | No included studies |
| VALUES | Important uncertainty or variability | Possibly important uncertainty or variability | Probably no important uncertainty or variability | No important uncertainty or variability | | | |
| BALANCE OF EFFECTS | Favors the comparison | Probably favors the comparison | Does not favor either the intervention or the comparison | Probably favors the intervention | Favors the intervention | Varies | Don't know |
| ACCEPTABILITY | No | Probably no | Probably yes | Yes | | Varies | Don't know |
| FEASIBILITY | No | Probably no | Probably yes | Yes | | Varies | Don't know |
